# Endothelial-derived cardiovascular disease-related microRNAs elevated with prolonged sitting pattern among postmenopausal women

**DOI:** 10.1038/s41598-021-90154-1

**Published:** 2021-06-03

**Authors:** Ya-Ju Chang, Fatima Tuz-Zahra, Suneeta Godbole, Yesenia Avitia, John Bellettiere, Cheryl L. Rock, Marta M. Jankowska, Matthew A. Allison, David W. Dunstan, Brinda Rana, Loki Natarajan, Dorothy D. Sears

**Affiliations:** 1 Department of Family Medicine, UC San Diego, La Jolla, CA USA; 2 Herbert Wertheim School of Public Health, UC San Diego, La Jolla, CA USA; 3 Center for Behavioral Epidemiology and Community Health, San Diego State University, San Diego, CA USA; 4 Moores Cancer Center, UC San Diego, La Jolla, CA USA; 5 Beckman Research Institute, City of Hope, Duarte, USA; 6 Baker Heart and Diabetes Institute, Melbourne, Australia; 7 Mary MacKillop Institute for Health Research, Australian Catholic University, Melbourne, VIC Australia; 8 Department of Psychiatry, UC San Diego, La Jolla, CA USA; 9 Department of Medicine, UC San Diego, La Jolla, CA USA; 10 College of Health Solutions, Arizona State University, 550 N 3rd Street, Phoenix, AZ 85004 USA

**Keywords:** Epidemiology, Translational research, Cell biology

## Abstract

Time spent sitting is positively correlated with endothelial dysfunction and cardiovascular disease risk. The underlying molecular mechanisms are unknown. MicroRNAs contained in extracellular vesicles (EVs) reflect cell/tissue status and mediate intercellular communication. We explored the association between sitting patterns and microRNAs isolated from endothelial cell (EC)-derived EVs. Using extant actigraphy based sitting behavior data on a cohort of 518 postmenopausal overweight/obese women, we grouped the woman as Interrupted Sitters (IS; N = 18) or Super Sitters (SS; N = 53) if they were in the shortest or longest sitting pattern quartile, respectively. The cargo microRNA in EC-EVs from the IS and SS women were compared. MicroRNA data were weighted by age, physical functioning, MVPA, device wear days, device wear time, waist circumference, and body mass index. Screening of CVD-related microRNAs demonstrated that miR-199a-5p, let-7d-5p, miR-140-5p, miR-142-3p, miR-133b level were significantly elevated in SS compared to IS groups. Group differences in let-7d-5p, miR-133b, and miR-142-3p were validated in expanded groups. Pathway enrichment analyses show that mucin-type O-glycan biosynthesis and cardiomyocyte adrenergic signaling (*P* < 0.001) are downstream of the three validated microRNAs. This proof-of-concept study supports the possibility that CVD-related microRNAs in EC-EVs may be molecular transducers of sitting pattern-associated CVD risk in overweight postmenopausal women.

## Introduction

In U.S. adults aged 45 years old or older, excessive sedentary behavior and prolonged sitting patterns are associated with obesity, cardiometabolic disorders, increased cardiovascular events, cancer, and all-cause mortality^1–3^. Sedentary behavior is characterized by energy expenditure lower than 1.5 metabolic equivalent (MET), including lying, reclining, and sitting^4^. On average, older adults (age ≥ 45 years) spend 65% of their waking hours in sedentary behavior, 33% in light physical activity (100–2019 counts per minute, cpm), and 2% in moderate-to-vigorous physical activity (MVPA, ≥ 1952 cpm), as measured with accelerometry^5,6^. Sitting is a non-movement posture form of sedentary behavior^4^. Using device-based measures of sitting behavior, we recently showed that longer total sitting time and mean sitting bout duration (i.e., sitting pattern) were associated with cardiometabolic and cancer risk biomarkers among 518 overweight and obese postmenopausal women^7^. In this population, the women had a mean (SD) daily sitting time of 9.1 (1.6) hours and a mean (SD) sitting bout duration of 39.2 (15.5) minutes. A nationwide study involving 4757 participants revealed women at mean age 47.7 spent an average 8.51 (1.29) hours in sedentary behavior and 5.46 (1.55) minutes in sedentary bouts^8^. Sitting and sedentary time are associated with CVD risk, including endothelial dysfunction.

Endothelial dysfunction is an early event during vascular injury, which impairs vasodilation and promotes development of plaque and inflammation in the vascular wall^9^. Several laboratory studies show that endothelial function is rapidly impaired by prolonged sitting^10,11^. Shortening bouts of prolonged sitting with low energy expenditure active breaks acutely prevents endothelial function decline in young, active adults and improves endothelial function in older, sedentary overweight/obese women^10,12,13^. Although the association between prolonged sitting and endothelial dysfunction are becoming clearer, the molecular mechanisms that link physiological alterations and pathological progression remain unknown.

In this regard, microRNAs (miRs) in endothelial cells (ECs) are one of the molecule classes that changes with cellular status in response to aging, oxidized low-density lipoprotein, hyperglycemia, hypertension, and magnitudes of antegrade blood flow^14,15^. MiRs are small non-coding RNAs of approximately 22 nucleotides, which alter biological functions by silencing genes via translational repression and mRNA degradation^16^. Growing evidence reveals that cargo molecules, including miRs, in extracellular vesicles (EVs) are stable markers for clinical diagnosis, prognosis, and monitoring treatment response^17^. In addition to regulating intra-cellular functions, miRs transported in EVs mediate tissue cross-talk and inter-cellular communication^18^. They are found in biofluids and can be biomarkers of the physiologic function and disease status of their originating cell type^18^. For example, EC-derived miR-92a secreted in CD144-enriched microparticles mediates endothelial dysfunction and thus predisposes chronic kidney disease patients to cardiovascular disease (CVD) progression^19^. Twelve miRs isolated from human plasma EVs have been identified to potentially impact muscle remodeling and growth resulting from acute exercise^20^.

Given that miRs may be biomarkers and molecular transducers of health outcomes and that the vascular endothelium is a primary tissue affected by prolonged sitting, we hypothesized that miR expression in EC-derived EVs (EC-EVs) are influenced by prolonged sitting pattern. Specifically, we examined whether miRs from isolated plasma EC-EVs used in a targeted screen of CVD-miRs are associated with sitting pattern among overweight/obese postmenopausal women with either short or long mean sitting bout duration. We further explored functional pathways through which the identified miRs may link the physiological perturbations and disease risk associated with prolonged sitting pattern.

## Results

From an original cohort of 518 postmenopausal, overweight or obese, sedentary women classified by a validated machine-learned algorithm based on accelerometer measures, 18 were categorized as Interrupted Sitters (IS) and 53 as Super Sitters (SS). The two groups were classified using quartile cross-tabulation of individual sitting patterns and MVPA, including the lowest and highest quartiles of mean sitting bout duration, and the lowest quartile MVPA (< 7 min/day, Supplementary Table S1). Data and plasma from these individuals were used to screen 84 CVD-related miRs (Supplementary Table S2) in the first stage of this study.

Table 1 shows participant demographic, activity, and cardiometabolic risk biomarker characteristics of the IS and SS groups. Although IS and SS are similar to each other in many respects, the SS group had significantly lower physical functioning, MVPA, and walking time, and greater total sitting time and mean sitting bout duration, compared to the IS group. SS women sat for an average of 11.0 ± 1.2 h per day in bouts averaging 64.0 min, while IS women sat for an average of 7.4 ± 1.1 h per day in bouts averaging 25.5 min.

**Table 1:** Demographics, activity-related measures, and cardiometabolic risk biomarkers of Interrupted Sitters (IS) and Super Sitters (SS).

	Total (n = 71)	IS (n = 18)	SS (n = 53)	*P*-value
**Age, mean (sd)**	65.9 (6.6)	64.2 (5.0)	66.5 (7.0)	0.12
**Race, n (%)**^a^				0.13
White	58 (81.7)	14 (87.5)	44 (83)	
Black	3 (4.2)	0 (0)	3 (5.7)	
Native American	1 (1.4)	0 (0)	1 (1.9)	
Asian	0 (0)	0 (0)	0 (0)	
Pacific Islander	2 (2.8)	2 (12.5)	0 (0)	
Other/Unknown	0 (0)	0 (0)	0 (0)	
Mixed	2 (2.8)	0 (0)	2 (3.8)	
**Hispanic ethnicity, n (%)**				0.42
Hispanic	24 (33.8)	1 (5.6)	9 (17)	
Non-Hispanic	47 (66.2)	17 (94.4)	44 (83)	
**Marital Status, n (%)**				0.12
Married/Living together	34 (47.9)	12 (66.7)	22 (41.5)	
Single/Divorced/Widowed/Separated	37 (52.1)	6 (33.3)	31 (58.5)	
**Highest education level, n (%)**				0.51
Up to high school completion	7 (9.9)	3 (16.7)	4 (7.5)	
Some college or vocation training	36 (50.7)	9 (50.0)	27 (50.9)	
College graduate	28 (39.4)	6 (33.3)	22 (41.5)	
**Physical functioning, mean (sd)**	61.2 (28.9)	74.9 (20.7)	56.5 (30.0)	0.01*
**Activity-related measures, mean (sd)**				
Total sitting time; min/day	603.4 (120.8)	442.6 (68.8)	658.0 (73.8)	< 0.01*
Mean sitting bout duration; min/day	54.3 (26.9)	25.5 (3.2)	64.0 (24.3)	< 0.01*
Moderate-to-vigorous activity; min/day	3.5 (1.8)	4.5 (1.6)	3.2 (1.8)	0.01*
Walking time; min/day	32.7 (25.8)	53.2 (30.7)	25.7 (19.9)	< 0.01*
**Cardiometabolic biomarkers, mean (sd)**				
Body mass index; kg/m^2^	32.8 (5.0)	31.3 (3.2)	33.3 (5.4)	0.06
Waist circumference; cm^b^	101.4 (15.9)	99.0 (8.5)	102.1 (17.7)	0.34
Fasting glucose; mg/dL^c^	116.3 (43.8)	120.4 (50.3)	115.0 (41.8)	0.68
Fasting insulin; pg/mL	671.9 (443.4)	644.4 (417.4)	681.3 (455.4)	0.75
HOMA-IR	5.6 (4.9)	5.3 (3.7)	5.8 (5.2)	0.68
HOMA2-IR	2.7 (1.9)	2.5 (1.5)	2.7 (2.0)	0.69
**Parent study, n (%)**				< 0.01*
CoM	21 (29.6)	2 (11.1)	19 (35.8)	
RFH	42 (59.1)	13 (72.2)	29 (54.7)	
MENU	8 (11.3)	3 (16.7)	5 (9.4)	

CoM, Community of Mine; RFH, Reach for Health; MENU, Metabolic, Exercise and Nutrition at University of California, San Diego; HDL, high-density lipoproteins; HOMA-IR, homeostatic model assessment of insulin resistance; LDL, low-density lipoproteins. *P*-values computed using Chi-square tests for categorical variables and t-tests for continuous variables.

*P < 0.05.

^a^Missing race data from 3 participants in RfH and 2 participants in CoM.

^b^Missing waist circumference data from 2 participants in RfH.

^c^4 participants in IS and 8 participants in SS had fasting glucose > 125 mg/dL.

In preparation for analysis of the clinical samples, we examined the specificity of CD144 for EC-EVs (vs. EVs from other cell types that predominate in the vasculature) and the biochemical and ultrastructural characteristics of the CD144 + EC-EVs. CD144, also known VE-cadherin, is a junctional protein specifically expressed on ECs for controlling vascular permeability. Dot-blot assay of EVs purified from conditioned media of ECs, Smooth muscle cells (SMCs), and peripheral blood mononuclear cells (PBMCs) showed CD144 protein signal on EC-EVs but not SMC- and PBMC-EVs (Supplementary Fig. S1A). CD63 protein, a common EV marker, was expressed on EVs from all three cell types. Transmission electronic microscopy (TEM) images with immunogold labeling show the ultrastructure of EVs and markers of EV (CD81, CD63, LAMP1) and EC (CD144) on EVs purified from EC-conditioned media and plasma (Supplementary Fig. S1B). Fluorescence flow cytometry analysis demonstrated a subpopulation of plasma CD63^+^ EVs co-stained with anti-CD144 antibody and the lipid membrane dye CFSE (Supplementary Fig. S1C). Approximately 71.1% of CD63^+^ EVs were CD144^+^CFSE^+^. Together, these experiments demonstrate that CD144 can be used to specifically identify EC-EVs.

To address the fraction and stability of miRs in intact EC-EVs, we conducted protection assays treating purified EC-EVs from EC-conditioned media with PBS, proteinase K (PK), Triton, and/or RNase. Levels of MiR-126, a known EC-enriched miR, were reduced 93% by the Triton + RNase treatment compared to the Mock treatment (PBS), suggesting that EV-contained miRs are protected from degradation in the circulation (Supplementary Fig. S1D). Treatment with RNase alone or with PK resulted in 24% and 31% reductions in miR-126 levels, respectively, suggesting that EC-EVs are intact and protect the majority of miRs from RNase degradation in the conditioned media.

Having developed a protocol to isolate EC-EVs from plasma, EC-EVs were isolated from IS or SS plasma samples using anti-CD144 immunoprecipitation and subsequent total RNA extraction. CVD-related miRs were screened for differential expression in IS and SS groups using an array of 84 CVD-related miRs. RNA samples were pooled in sets of 3 (a single SS pool consisted of only 2 samples), as shown in Supplementary Fig. S2. Obtained miR expression values across the samples were adjusted by balancing on key covariates using Inverse Probability of Treatment Weighting (IPTW). MiR-133b, miR-140-5p, miR-142-3p, let-7d-5p, miR-199a-5p were selected for technical validation using a two-step process. The top 10 miRs were identified by standardized absolute ATE ranking. Then, in that set of 10 miRs, the 5 miRs with the largest standard deviation were selected (Table 2) to ensure the low SD did not artificially inflate the ATE, and hence lead to a high ATE rank in the first step. Supplementary Table S3 shows standardized absolute ATE rankings of the other 74 miRs on the array.

**Table 2:** Top 10 miRs ranked by standardized average treatment effect (ATE) & standard deviation (SD) of miR levels from IS and SS groups.

	Mean difference in relative expr	Relative expr, SD^a^	Standardized ATE rank	Standardized ATE	SD rank
miR-133b*	11.30	5.15	7	4.38	31
miR-140-5p*	5.70	2.26	8	4.37	43
miR-142-3p*	5.15	2.21	3	5.29	45
let-7d-5p*	4.19	1.94	2	5.33	53
miR-199a-5p*	3.25	1.53	1	5.44	58
miR-146a-5p	2.96	1.51	5	4.82	60
miR-103a-3p	1.80	0.80	10	4.17	70
miR-424-5p	1.15	0.61	6	4.50	75
let-7e-5p	0.52	0.28	9	4.20	80
miR-155-5p	0.76	0.27	4	5.02	81

ATE, average treatment effect: weighted mean difference of miR levels between SS and IS; Standardized ATE, ATE divided by bootstrapped SD of ATE; Expr, expression. SD, standard deviation.

*Top 5 miRs based on combined standardized ATE ranking then, SD ranking.

^a^Unweighted SD across IS and SS.

We next conducted technical validation studies of the 5 prioritized miRs, miR-133b, miR-140-5p, miR-142-3p, let-7d-5p, miR-199a-5p, using individual EC-EV RNA samples. In addition, we augmented the IS and SS groups for Hispanic ethnicity to enable exploration of potential differences by ethnicity in miR expression associated with IS and SS sitting patterns. All women who self-identified as Hispanic and who were in the 2nd-lowest quartile of MVPA (7–15.5 min/day) and 1st and 4th quartiles of mean sitting bout duration (N = 7 from each) were added to the original IS and SS groups to create the amended groups IS + (n = 25; 8 Hispanic) and SS + (n = 60; 16 Hispanic), respectively (Supplementary Table S4). These amended groups were used to validate the 5 miRs selected in the screening stage of analyses.

**Figure 1:**
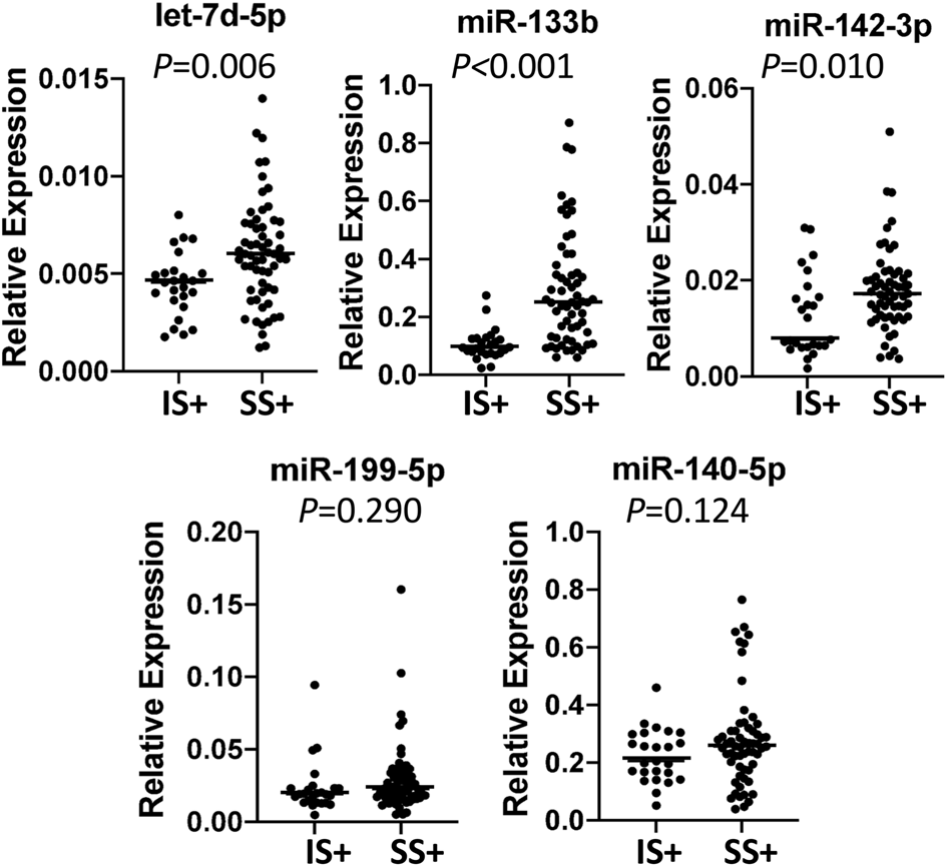
Differential expression of individually validated miRs from IS + and SS + (unadjusted data). The highest ranking 5 cardiovascular disease (CVD)-related EC-EV miRs initially identified in pooled RNA sample sets from IS and SS were validated by qPCR in individual RNA samples. IS + : Interrupted Sitter group with enhanced ethnic diversity (N = 25); SS + : Super Sitter group with enhanced ethnic diversity (N = 60). Statistical significance between IS + and SS + groups was examined by unadjusted t-test. Horizonal bars indicate group means.

**Figure 2:**
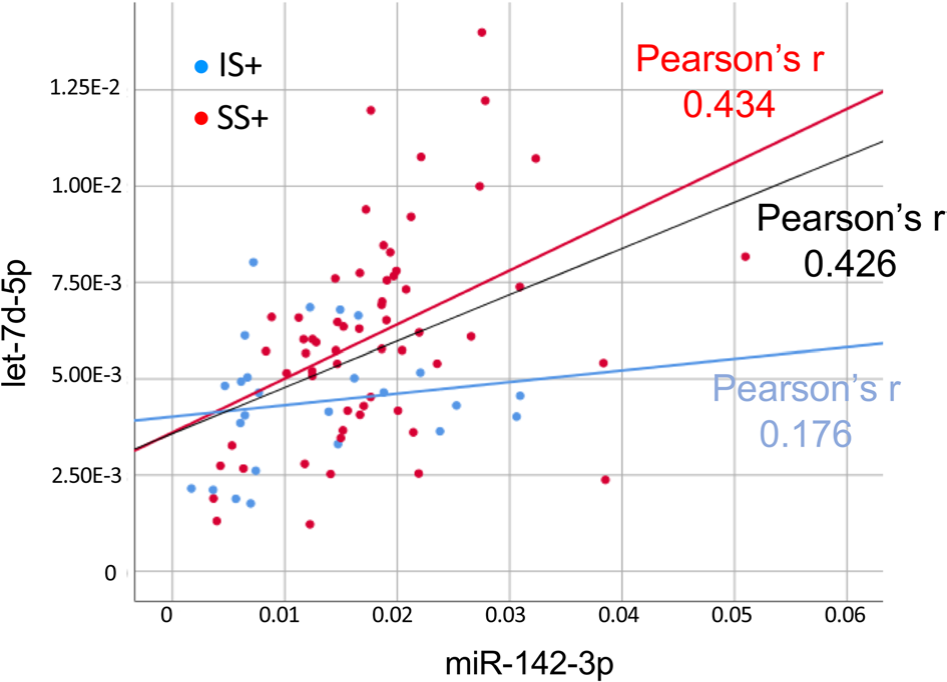
Correlation between miR-142-3p and let-7d-5p. Measurements of miR-142-3p and let-7d-5p from individual participants in IS + and SS + were plotted. Data from participants in IS + and SS + groups are shown in blue and red dots, respectively. Blue, red, and black lines indicate linear correlations in IS + , SS + , and combined groups, respectively.

Supplementary Table S4 shows the quartile characterization of mean sitting bout duration and MVPA for the IS + and SS + groups. Supplementary Table S5 shows the age and activity-related characteristics. Differential expression was confirmed for let-7d-5p, miR-133b, and miR-142-3p using unadjusted data, with greater miR expression in EC-EVs from SS + compared to IS + individuals (Fig. 1). Differential expression of let-7d-5p, miR-133b, and miR-142-3p between IS + and SS + was sustained after IPTW (Table 3). We examined correlations among miRs using unadjusted data (Supplementary Table S6). Let-7d-5p and miR-142-3p were positively correlated (Pearson’s *r*: 0.426) across all IS + and SS + individuals. After group stratification, we found a stronger correlation between let-7d-5p and miR-142-3p in SS + (Pearson’s *r*: 0.434) than IS + (Pearson’s *r*: 0.176, Fig. 2).

**Table 3:** Validation analysis of target miRs differentiating IS + and SS + groups.

	ATE	95%CI	Standardized ATE
miR-133b	0.168	(0.108, 0.228)*	5.462
let-7d-5p	0.002	(0.001, 0.004)*	3.323
miR-142-3p	0.006	(0.001, 0.011)*	2.196
miR-140-5p	0.056	(− 0.026, 0.138)	1.332
miR-199-5p	0.010	(0.000, 0.0206)	1.973

ATE, average treatment effect: weighted mean difference of miR levels between SS + and IS + ; Standardized ATE, ATE divided by bootstrapped SD of ATE.

*Statistically significant, zero not included in 95% CI.

To explore possible miR-mediated biological mechanisms that underlie associations between prolonged sitting pattern and cardiometabolic risk in older women, we conducted bioinformatics-based functional pathway analyses on the 3 validated miRs (let-7d-5p, miR-133b, and miR-142-3p). Two approaches were used: TargetScan to predict miR target genes based on potential sequence alignments between the miRs and target mRNAs; and TarBase to identify miR target genes supported by experimental evidence in the literature. Pathway analysis using TargetScan identified 7 functional pathways as potential targets of let-7d-5p, miR-133b, and/or miR-142-3p: mucin type O-Glycan biosynthesis, adrenergic signaling in cardiomyocytes, signaling pathways regulating pluripotency of stem cells, valine, leucine, and isoleucine biosynthesis, biosynthesis of amino acids, oocyte meiosis, and adherens junction (Table 4). Pathway analysis using TarBase identified 20 functional pathways as potential targets of let-7d-5p, miR-133b, and/or miR-142-3p (Table 4, Supplementary Tables S7 and S8). Direct and indirect target genes of let-7d-5p and miR-143-3p that are components of the adherens junction pathway are shown in Supplementary Fig. S3. This pathway is a potential mechanism underlying prolonged sitting among overweight/obese postmenopausal women.

**Table 4:** Functional pathway analysis of predicted sequence and literature-based miR target genes.

KEGG pathway^a^	Mucin type O-Glycan biosynthesis	Adrenergic signaling in cardiomyocytes	Signaling pathways regulating pluripotency of stem cells	Valine, leucine and isoleucine biosynthesis	Biosynthesis of amino acids	Oocyte meiosis	Adherens junction
*P*-value	2.42E−08	4.34E−04	3.70E−03	4.51E−03	3.24E−02	4.05E−02	4.48E−02
MicroRNAs associated with prolonged sitting	miR-133b let-7d-5p	miR-133b	let-7d-5p	let-7d-5p	let-7d-5p miR-142-3p	miR-133b let-7d-5p miR-142-3p	miR-142-3p
Predicted target genes (direct target)	*GALNT1*	*PPP2CA*	*ACVR1C*	*BCAT1*	*ARG2*	*CDC25C*	*RAC1*
	*GALNT8*	*PPP2R2D*	*HAND1*		*BCAT1*	*CPEB1*	*WASL*
		*PPP2CB*	*HOXB1*		*TKTL2*	*ITPR2*	
		*TPM4*	*SKIL*			*PPP2CA*	
			*SMARCAD1*			*PPP2CB*	
			*WNT9A*			*SMC1A*	

KEGG Pathway^b^	Adherens junction	Lysine degradation	TGF-β signaling pathway	Hippo signaling pathway	Viral carcinogenesis	Cell cycle	Pathways in cancer
*P*-value	5.60E−07	9.75E−06	2.66E−05	9.22E−05	1.09E−04	1.19E−04	1.68E−04
MicroRNAs associated with prolonged sitting	let-7d-5p miR-142-3p	let-7d-5p miR-142-3p	let-7d-5p miR-142-3p	let-7d-5p miR-142-3p	miR-133b let-7d-5p miR-142-3p	let-7d-5p	let-7d-5p miR-142-3p
Literature-supported target genes (direct target)	*ACTG1*		*CREBBP*	*ACTB*	*CDK4*	*CCNA2*	*AR*
	*CREBBP*		*E2F4*	*ACTG1*	*NRAS*	*CCND1*	*ARHGEF1*
	*CTNNB1*		*E2F5*^c^	*BIRC2*	*YWHAE*	*CCNE2*	*BRAF*
	*FGFR1*		*MAPK1*	*CCND1*	*CCNA2*	*CDK4*	*CASP3*
	*IGF1R*		*MYC*	*CTNNB1*	*ATF6B*	*CHEK1*	*CDK4*
	*MAPK1*		*PPP2CA*	*DVL3*	*YWHAG*	*CREBBP*	*CCND1*
	*PTPN6*		*PPP2CB*	*FZD1*	*DDX3X*	*E2F2*	*CCNE2*
	*PTPRJ*		*PPP2R1B*	*FZD3*	*CHEK1*	*E2F3*	*CXCR4*
	*RAC1*		*RPS6KB2*	*GLI2*	*TP53*	*E2F4*	*DVL3*
	*SMAD2*		*SMAD2*	*MYC*	*CASP3*	*MDM2*	*E2F2*^c^
	*SMAD3*		*SMAD3*	*PPP2CA*	*CCND1*	*MYC*	*E2F3*
	*SMAD4*		*SMAD4*	*PPP2CB*	*CCNE2*	*SMAD2*	*FADD*
	*TGFBR1*^c^		*SMAD7*	*PPP2R1B*	*SKP2*	*SMAD3*	*FZD3*
	*TGFBR2*		*TGFBR1*^c^	*PARD6B*	*CCR5*	*SKP2*	*GLI2*
			*TGFBR2*	*SMAD2*	*PRKACA*	*TP53*	*HIF1A*
				*SMAD3*	*DDB1*	*YWHAE*	*HSP90AA1*
				*SMAD4*	*RAC1*	*YWHAG*	*IGF1R*
				*SMAD7*	*CDKN1A*		*ITGAV*
				*TGFBR1*	*MAPK1*		*MAPK8*
				*TGFBR2*	*CREBBP*		*MSH6*
				*YWHAE*	*JAK1*		*NRAS*
				*YWHAG*	*MDM2*^c^		*PLCG1*
							*RUNX1*
							*SMAD2*
							*SMAD3*
							*SKP2*
							*TGFBR1*^c^
							*TP53*
Literature-supported target genes (non-direct target)	*ACTB*	*SETD1B*	*ACVR1B*	*PPP1CA*	*PKM*	*ESPL1*	*ADCY1*
	*ACP1*	*PLOD2*	*ACVR2A*	*YAP1*	*HLA-B*	*CDC6*	*ETS1*
	*CSNK2A1*	*KMT2D*^c^	*BAMBI*	*AMOT*	*DDX2X*	*PCNA*	*GNG12*
	*CSNK2A2*	*SUV420H1*	*PPP2R1A*	*LIMD1*	*EP300*	*CCNB1*	*LPAR2*
	*CTNND1*	*DOT1L*^c^	*BMP8A*	*CSNK1E*	*GTF2B*	*ORC2*	*GNB1*
	*FARP2*	*KMT2C*	*BMPR2*	*TEAD4*	*MRPS18B*	*DBF4*	*ARNT2*
	*FER*	*SETD1A*		*MOB1A*	*HPN*	*STAG2*	*GNB2*
	*FYN*	*EHMT1*		*FHF1*	*HIST1H2BG*	*WEE1*	*MYC*
	*INSR*	*KMT2B*		*WNT9A*	*RBPJ*	*E2F5*	*RASGRP4*
	*NLK*	*WHSC1L1*		*FZD7*	*HIST1H2BL*	*CDC45*	*PDGFB*
	*PTPRF*	*ASH1L*		*PPP2R2C*	*GTF2A1*	*CDC27*	*PRKACA*
	*PVRL2*	*WHSC1*		*PPP2R2A*	*SRF*	*CDKN1A*	*EGLN3*
	*SSX2IP*	*KMT2A*		*PPP2R1A*	*GTF2E1*	*PRKDC*	*RAC1*
	*TJP1*	*ALDH9A1*		*CSNK1E*	*HIST1H2BB*	*PLK1*	
	*VCL*	*PLOD1*		*BMP8A*	*HIST1H4D*	*ANAPC13*	
	*WASF1*			*SMAD7*	*ATF2*	*MCM3*	
	*WASF2*			*LATS1*	*GTF2H1*	*CDC25A*	
	*WASL*			*BMPR2*	*CREB5*		
	*YES1*				*PIK3R2*		

^a^KEGG pathways were identified based on TargetScan predicted miR target genes.

^b^KEGG pathway analysis based on Tarbase v7.0

^c^Genes targeted by 2 miRs.

## Discussion

In this exploratory, proof of concept study, sitting patterns consisting of longer mean bout duration (SS + ; 62.8 ± 23.4 min) were associated with significant elevation of let-7d-5p, miR-133b, and miR-142-3p in circulating EC-EVs, compared to patterns with more interrupted, shorter sitting bouts (IS + ; 25.9 ± 2.8 min) among 85 overweight/obese postmenopausal women. Pathway analyses of the putative and literature supported gene targets of these three miRs suggest that they may alter multiple biological pathways relevant to sitting time-associated disease risk. To our knowledge, this is the first study to demonstrate a link between device-measured sitting pattern differences and EC-originating, circulating miRs in any population. It is also the first to identify candidate molecular mediators of health impacted by sitting pattern.

Adults who are older than 60 years are the largest sedentary population in the United States^21^. Give the association of sedentary time and CVD risk, this risk may be augmented in postmenopausal women who have higher CVD risk due to steep estrogen decline during menopause^22^. Although older women spend less time on sedentary behavior than age-matched men, CVD risk driven by biological differences and lifestyle factors are notable among this high-risk population^21^. Dose–response associations of CVD risk with sitting time and patterns was demonstrated in a large cohort study among older women (age range 63–97 years)^2^. Postmenopausal women have increased risk for overweight/obesity and insulin resistance, which further elevates CVD risk^23^. Clinical and epidemiological evidence indicate that research studies among postmenopausal women is urgently needed to enhance healthy aging and quality of life for this special population. The women in the SS group on average sat 3.6 h longer per day and spent 38.5 min longer at each sitting bout than the women in the IS group (Table 1). Daily behavior is composed in an isotemporal framework wherein reduction in sitting time is simultaneously associated with an increase in other non-sitting behaviors. There was a 1.3-min difference in daily MVPA and 27.5-min difference in daily walking time between IS and SS women, suggesting that “interrupted sitter” overweight/obese postmenopausal women replace sitting behavior with light physical activity (i.e., walking time) rather than MVPA (Table 1). Daily MVPA in the combined groups was very low (3.5 ± 1.8 min/day; mean ± SD). Although the 1.3-min group difference in daily MVPA was statistically significant, it is unlikely to be associated with a biologically or clinically significant impact.

During prolonged sitting, particularly in uninterrupted bouts, blood flow and skeletal muscle contractions are reduced in the lower extremities, collectively contributing to prolonged sitting-associated endothelial dysfunction^12,24^. Expression of miRs in ECs is dynamic and dose-responsive to reflect endothelial homeostasis or dysfunction in response to external stimuli and conditions such as those associated with prolonged sitting^25,26^. The three miRs identified in this study are linked with CVD risk factors and EC biology. Overexpression of miR-133b in human retinal EC exposed to a hyperglycemic condition prohibited proliferation and facilitated apoptosis^27^. Overexpression of miR-142-3p in primary human aortic endothelial cells prevented high-glucose-induced endothelial-to-mesenchymal transition, a process involved in cardiac fibrosis^28^. Systemic administration of let-7d mimetics into diabetic ApoE^-/-^ mice decreased inflammatory genes, suggesting a protective role of let-7d in diabetes-associated atheroscleorsis^29^. In an atherosclerotic mouse model with chronic inflammation, miR-133b and miR-142-3p facilitated vulnerable plaque formation and induced EC apoptosis, respectively^30,31^. Let-7d overexpression was shown to inhibit endothelial migration, proliferation, and angiogenesis in vitro^32^. These studies demonstrate that potential EC regulatory function and CVD-causal effects of the three differentially expressed miRs identified in the present study are associated with the prolonged sitting pattern.

Extracellular miRs released from cells and tissues are potential biomarkers and/or mediators of acute myocardial infarction, chronic heart failure, diabetes, and other CVDs to mediate cellular communication when they are taken up by other cells^17,33–35^. Body fluid levels of extracellular miRs, without tissue(s) origin identity, are associated with modifiable lifestyle factors, including screen time (a sedentary behavior-associated activity), exercise, and diet^36–38^. Among 80 primary school children in Belgium (COGNition and Air pollution in Children study), each additional screen time hour per week was associated with a 3.44% higher level of miR-222 and 1.84% higher level of miR-146a in saliva^36^. Regular exercise for 20 weeks significantly increased miR-142-3p, miR-221-3p, miR-126-3p, miR-146-5p, and miR-27b-3p, and decreased miR-486-5p, let-7b-5p, miR-29c-3p, let-7e-5p, miR-93-5p, miR-7-5p, miR-25-3p, miR-92a-3p, and miR-29b-3p in serum from 20 participants enrolled in the HERITAGE Family study^37^. Nine miRs, including miR-10b, miR-155, miR-200b, miR-296-5p, miR-375, miR-92a, miR-145, miR-204, and miR-211, responded to dietary zinc deprivation and repletion among 10 men from the General Clinical Research Center at University of Florida^38^. We found that older women who spent an average of 62.8 ± 23.4 uninterrupted minutes in a sitting posture (mean sitting bout duration) have a higher level of let-7d-5p, miR-133b, and miR142-5p in circulating EC-EVs compared to those who sat 25.9 ± 2.8 min.

By targeting EVs with specific tissue origin, this study provides a better resolution of physiological-to-pathological changes in endothelial dysfunction. It identifies potential sitting behavior-associated miR target signaling pathways in ECs as well as recipient cells and tissues that mediate sitting-associated disease risk. Functional pathway enrichment analyses of the top three differentially expressed, technically validated miRs identified target genes and biological pathways. Prolonged sitting patterns among postmenopausal women potentially affect (1) cellular and vascular function through regulating mucin-type O-glycosylation and adherens junction pathways, (2) cardiomyocyte function via modulation of adrenergic signaling, and (3) branched chain amino acid (BCAA; valine, leucine, and isoleucine) metabolism. Mucin-type O-glycosylation is a glycosylation type that adds an N-acetylgalactosamine moiety to serine and threonine residues in proteins and these glycosylation modifications are critical for vascular integrity, especially during blood vessel development^39^. Adherens junctions are the major structural components that create cell-to-cell barriers and enable endothelial cells to control vascular permeability^40^. Abnormal β-adrenergic signaling usually found in aged hearts with cardiac dysfunction^41^. Elevation of circulating BCAA concentrations is associated with insulin resistance, onset of type 2 diabetes and cardiovascular events, and mitochondrial dysfunction^41–43^, and BCAA concentrations are decreased with weight loss and insulin sensitization^42,43^.

In this exploratory study, we identified three EC-derived, circulating miRs that bridge device-measured sitting patterns to biological effector genes and pathways. Several limitations warrant acknowledgement when interpreting our findings. First, the small sample size of this study may limit power to detect statistical differences, and/or increase the risk of spurious findings. Second, dietary factors have been found to slightly attenuate the association between TV-watching sedentary behavior and obesity in women^44^. Thus, dietary patterns and other unmeasured covariates could contribute to residual confounding that we were unable to control for in our models. Third, we relied on data from hip-worn accelerometer devices to measure sitting, which could misclassify standing without ambulation as sitting. However, we processed the accelerometer data using a machine learning technique that was specifically designed to distinguish between sitting and standing^45,46^. Despite this, miss-classification can still occur, and future studies would benefit from using devices such as an inclinometer, combinations of devices, or data processing techniques that can more accurately distinguish between sitting and standing. Fourth, our current study outcomes in a high-risk population (overweight/obese postmenopausal women) may not be generalizable. Future studies with larger scale and continuous analysis are needed across different sitting pattern compositions. Our results shed light on the underlying tissue-specific mechanisms linking sedentary behavior/sitting time and cardiometabolic health in older women. The predictive value of EC-originating, circulating miRs for sitting time and/or sitting-associated disease risk needs to be corroborated by interventional and longitudinal studies.

In conclusion, our results demonstrate that the EC-EVs carrying CVD-related miRs may be a relevant indicator for evaluating prolonged sitting patterns and cardiometabolic health risk in postmenopausal overweight or obese women. Longitudinal cohort studies and field-based randomized control trials exploring whether circulating CVD-related miRs change in response to sitting time reduction, particularly prolonged sitting patterns, are needed to elucidate the potential causality of CVD-related miRs on sedentary behavior-associated disease risk.

## Methods

The data that support the findings of this study are available from the corresponding author. All methods were performed in accordance with the relevant guidelines and regulations of University of California, San Diego (UCSD).

### Participants and study design

This is a cross-sectional study that explored sitting pattern-associated miRs contained in circulating EC-EVs. Archival data and samples from a previously described cohort of 518 postmenopausal overweight/obese women, minimum 55 years of age and body mass index (BMI) of 25 kg/m^2^^7^, were used to identify a subset of women with low MVPA and short or long sitting pattern and conduct analyses of circulating miRs. Postmenopausal status of each participant was defined by age over 55 years old. Participants in the source cohort were originally enrolled in one of three clinical studies: Community of Mine (cross-sectional study)^47^, The Metabolism, Exercise and Nutrition at UCSD (MENU) study (randomized control trial)^48,49^, and The Reach for Health study (randomized control trial)^50^. These three studies have undergone review and approval through the UCSD Institutional Review Board.

All participants provided written informed consent. Data and plasma samples used in the current study were collected either from the single Community of Mine clinic visit or the baseline time points of the two randomized control trials mentioned above. Data from all 3 studies were critical for enrollment of participants, which resulted in low missingness (see footnote in Table 1).

### Activity measurement and sitting pattern classification

Activity measurement and sitting pattern characterization were described previously^7^. Briefly, all participants from the three studies wore an accelerometer (ActiGraph GT3X +) on their right hip for at least 4 days and up to 14 days, depending on the parent clinical study. Participants with less than 7 days of data (approximately 19% of the total) had at least one weekend day of wear time. The device was only removed when sleeping, showering, or swimming. Sitting posture was determined using raw accelerometer data that were processed by applying a previously validated machine-learned algorithm that was developed and validated specifically for older women using data collected from camera’s worn around participants neck for up to 7 days during free-living behavior^45,46^. The algorithm classified each minute of participants’ days into sitting, standing, daily life movement, and walking, and in the present analysis, we focused exclusively on the sitting compartment. Non-wear time was identified using the commonly used Choi algorithm^51^. Total sitting time was measured as the mean time spent sitting across all adherent days (i.e., days with at least 10 h of wear time)^52^. Consecutive minutes spent sitting were classified as sitting bouts (with no minimum duration and no tolerance), and the arithmetic mean of sitting bout durations were used to measure patterns of sedentary behavior. MVPA was calculated as minutes per day using accelerometer data and the commonly-used cut point of ≥ 1952 cpm^53^.

### Cardiometabolic biomarkers and physical functioning

Cardiometabolic biomarker (BMI, waist circumference, fasting glucose, fasting insulin, homeostatic model assessment of insulin resistance index (HOMA-IR), and HOMA2-IR) and physical functioning measurements were collected from all three parent studies, as described previously^7^. Fasting blood from participants was collected using EDTA vacutainers, followed by plasma isolation using centrifugation, and sample storage at -80 °C. All the EDTA-plasma samples are similar in age/time since draw across the three parent studies. Some of these cardiometabolic biomarker and physical functioning measures were used as analysis covariates and all were tabulated to characterize the population’s cardiometabolic risk (Table 1). Self-report of type 2 diabetes was an exclusion criterion for the MENU and Reach for Health studies. Type 1 diabetes was an exclusion for all three parent studies. Ten participants from Community of Mine included in the current analyses reported having type 2 diabetes. Twelve participants had fasting glucose levels in the type 2 diabetes range of > 125 mg/dL (Table 1 footnote).

### EC-EV characterization

*EV surface marker analysis*. Conditioned media from cultured human umbilical venous endothelial cells (ECs), human umbilical artery SMCs, and PBMCs was collected after incubation at 37 °C for 2 days. Growth media included 10% fetal bovine serum and 1% penicillin and streptomycin. EVs were collected from the media by centrifugation for 10 min at 2000 rcf at 4 °C to remove cell debris, followed by 90 min of ultracentrifugation at 110,000 rcf at 4 °C. EV pellets were resuspended in PBS and the ultracentrifugation step was repeated^54^. EVs from ECs, SMCs, and PBMCs were blotted onto a nitrocellulose membrane loading equal amounts of protein (0.5 µg). Primary antibodies specific for CD63 (Santa Cruz, sc-365604) and CD144 (Santa Cruz, sc-52751), appropriate secondary antibodies (Santa Cruz), and enhanced chemiluminescence HRP substrate system (Thermo Fisher Scientific, 32,109) were used for signal detection. *Transmission electron microscopy*. Immunogold labeling of ultracentrifugation-purified EC-EVs was performed by fixing EV samples with 4% paraformaldehyde onto electron microscopy grids before blocking and incubating with primary antibodies against CD81 (Santa Cruz, sc-7637), CD63 (Santa Cruz, sc-365604), LAMP1(Abcam, Ab24170), and CD144 (Santa Cruz, sc-52751)^55^. Appropriate secondary antibodies conjugated with 12 nm gold (Jackson ImmunoResearch) were hybridized onto EV samples and the samples stabilized with 1% glutaraldehyde. After air-drying, image capture of the samples was conducted at UC San Diego Electronic Microscopy Facility using a JEOL 1200 EXII transmission electron microscope with a 35 mm port digital camera. *MiR protection assays.* Experiments were conducted by using EC-EVs isolated from conditioned media. PBS (Mock) and RNase A (10 µg/mL) samples were incubated for 15 min at 37 °C. RNase A (10 µg/mL) + Proteinase K (PK, 20 µg/mL) samples were treated with proteinase K for 15 min at 55 °C and then incubated with RNase A for 15 min at 37 °C. RNase A (10 µg/mL) + Triton X-100 (Triton, 5%) samples were treated with Triton for 15 min at room temperature and then incubated with RNase A for 15 min at 37 °C. *Flow cytometry*. Samples for flow cytometry were prepared by combining 30µL plasma and 70 µL of PBS + 0.1% bovine serum albumin (BSA), followed by incubation with pre-washed anti-CD63-conjugated Dynabeads™ (ThermoFisher Scientific, 10606D) at 4 °C overnight on a flip-table. The plasma-Dynabead™ mixture was incubated with a membrane dye, carboxyfluorescein diacetate succinimidyl ester (CFSE; ThermoFisher Scientific, C34554), at 37 °C for 2 h then CD144 antibody conjugated with PE fluorophore (eBioscience™, 12-1449-80) at room temperature for 15 min. Beads bound with EVs were washed three times with 150uL PBS + 0.1% BSA before loading into the flow cytometer (BD Accuri™ C6).

### Plasma EC-EV isolation and RNA extraction

CD144^+^ EVs were immunoprecipitated from 0.5 mL archival plasma (Supplementary Fig. S2A). Plasma samples were diluted 1:1 with PBS + 0.1% BSA and incubated with 5 µg anti-CD144 antibody (Santa Cruz, sc-52751) at 4 °C overnight on a flip-table. Samples were then incubated with 100 µL pre-washed Dynabeads™ M-280 conjugated with anti-mouse IgG (ThermoFisher Scientific, 11202D) for 2 h at room temperature. After precipitation, Dynabeads™ M-280 with EV binding were washed once with 200uL PBS + 0.1% BSA. Synthetic *Caenorhabditis elegans* miR-39 (Cel-miR-39, 10 pmol; Qiagen) was added into each sample as a spike-in control before RNA isolation. RNA was extracted and purified from the washed beads using the miRNeasy kit (Qiagen). RNA was concentrated using 100µL 100% ethanol and incubation at − 20 °C for 1 h, followed by 12,000 rcf centrifugation at 4 °C for 30 min. RNA pellets were washed with 75% ethanol and dissolved in nuclease free water for further analysis.

### Real-time reverse transcriptase (RT)-polymerase chain reaction (PCR) array and individual sample validation

In the first stage of analyses, EC-EV miRs were screened by profiling pooled RNA samples from the IS and SS groups (Supplementary Fig. S2B) and using the miScript II RT Kit and Human Cardiovascular Disease miScript miRNA PCR Array (Qiagen, MIHS-113Z) which includes 84 CVD-related miRs. Each sample pool included 6 ng of total RNA from 3 individuals from the same group (IS or SS), with one sample per pool from each of the three parent studies when possible (otherwise random). Due to the number of women in the SS group not being divisible by 3 (n = 53, Fig. S2B), one pooled sample from this group included samples from only 2 individuals. In the second stage of the analyses, 5 miRs, miR-199a-5p, let-7d-5p, miR-140-5p, miR-142-3p, and miR-133b, were selected for validation of differential expression using individual RNA samples from the IS + and SS + groups. RNA samples were analyzed using miR-specific miScript Primer Assays (Qiagen) and miScript SYBR Green PCR Kit (Qiagen, 218,075). Relative expression of EC-EV miRs was calculated by normalizing CVD miRNA signals to the mean signal from the Qiagen array control panel (1st stage, screening) or SNORD61 and cel-miR-39 (2nd stage, validation), per manufacturer protocol. Supplementary Table S9 shows catalog number details of Primer Assays for the 5 miRs and controls for validation study.

### Pathway enrichment analysis

Pathway enrichment analyses were conducted on the three individual-sample validated miRs (let-7d-5p, miR-142-3p, and miR-133b). DIANA-miRPath v.3 was used to predict miR:gene interactions, functional characterization, and pathway enrichment of the 3 target CVD-related miRs by incorporating Kyoto Encyclopedia of Genes and Genomes (KEGG) pathways with TargetScan or Tarbase v7.0^56^. Supplementary Table S10 shows target genes of the 3 miRs. The web server ranked the biological pathways according to the enrichment analysis *P*-value of Fisher’s Exact Test.

### Statistical analysis

This study aimed to profile EC-EV miRs that were differentially expressed between pooled IS and SS group samples and then validate 5 of the miRs in individual samples from the IS + and SS + groups. Demographics, activity-related measures, and cardiometabolic-risk biomarkers data of IS and SS groups were compared using t-tests for continuous variables and chi-squared tests for categorical variables. A threshold α-level of 0.05 was used as the criterion to define statistically significant differences between groups. The strength of correlation was measured by Person’s correlation coefficient.

Inverse probability of treatment weighting (IPTW)^57^ was applied to the miR data (from pooled and individual samples) to balance effects of imbalanced participant covariates that might confound identification of sitting time-associated miRs. IPTW included physical functioning score, MVPA, number of device wear days, device wear time, waist circumference, BMI, and age, which could technically and biologically explain (confound) associations between sitting time and miRs^58,59^. Weights for IPTW were estimated using covariate balancing propensity score (CBPS) methodology and stabilized weights were used to address possible extreme values in weights^60,61^. Covariate balance after weighting was quantitatively assessed by comparing means, higher order moments and interactions using weighted standardized average differences, and comparing distributions of confounders using the Kolmogorov–Smirnov test statistic^57^. After IPTW application, all the covariates in IS vs. SS and IS + vs. SS + had absolute standardized average differences of less than 27% (Supplementary Table S11).

Average treatment effects (ATEs) were calculated by a weighted mean difference of miR levels between SS versus IS and SS + versus IS + . The standard deviations of ATE were calculated using bootstrapping-variability from the propensity score model was accounted for within the bootstrap process. The top 10 of the 84 possible CVD-related miRs on the array were ranked by standardized absolute ATE (ATE divided by the bootstrapped standard deviation) to begin selection of the five best miR candidates to move forward to validation analyses. Of the resulting 10 high-ranking miRs, those 5 with very low unweighted standard deviation compared to the other 5 miRs were eliminated as validation candidates. MiRs with relatively low unweighted standard deviation had smaller variance in expression across the IS and SS groups and, thus, were likely to artificially inflate the ATE. Statistical significance of differential miR expression between IS + and SS + was evaluated using bootstrapped confidence intervals. Interaction between sitting patterns and Hispanic ethnicity was assessed using weighted linear regression. Single data imputation was done using multiple imputations by chained equation, using predictive mean matching for continuous variables^62^.

## Supplementary Information

Supplementary Information

## Data Availability

The datasets generated during and/or analyzed during the current study are available from the corresponding author on reasonable request.
