# Supplementary material for: Endothelial-derived cardiovascular disease-related microRNAs elevated with prolonged sitting pattern among postmenopausal women
Source: Sci Rep. 2021 Jun 3;11:11766. doi: 10.1038/s41598-021-90154-1 (PMC8175392; doi:10.1038/s41598-021-90154-1)
Supplement: Supplementary file 1 — Supplementary Information [file 41598_2021_90154_MOESM1_ESM.pdf]

SUPPLEMENTAL INFORMATION

Endothelial-derived cardiovascular disease-related microRNAs elevated with prolonged sitting pattern among postmenopausal women

Ya-Ju Chang, PhD[1]; Fatima Tuz-Zahra, MS[2]; Suneeta Godbole, MPH, MS[1]; Yesenia Avitia, BS[1]; John Bellettiere, MA, MPH, PhD[2,3]; Cheryl L. Rock, PhD, RD[1,4]; Marta M. Jankowska, PhD[5]; Matthew A. Allison, MD, PhD[1]; David W Dunstan, PhD[6,7]; Brinda Rana, PhD[4,8]; Loki Natarajan, PhD[2,4]; and Dorothy D. Sears, PhD[1,4,9,10]

1 – Department of Family Medicine, UC San Diego, La Jolla, CA, USA

2 – Herbert Wertheim School of Public Health, UC San Diego, La Jolla CA, USA

3 – Center for Behavioral Epidemiology and Community Health, San Diego State University, San Diego, CA, USA

4 – Moores Cancer Center, UC San Diego, La Jolla CA, USA

5 - Beckman Research Institute, City of Hope, Duarte USA

6 – Baker Heart and Diabetes Institute, Melbourne, Australia

7 – Mary MacKillop Institute for Health Research, Australian Catholic University, Melbourne, Victoria, Australia

8 – Department of Psychiatry, UC San Diego, La Jolla CA, USA

9 – Department of Medicine, UC San Diego, La Jolla, CA, USA

10 – College of Health Solutions, Arizona State University, Phoenix, AZ, USA

Supplementary Tables

Table S1. Identification of Interrupted Sitters (IS) and Super Sitters (SS) based on quartiles of mean sitting bout duration and moderate-to-vigorous physical activity (MVPA)

MVPA (min/day)	Mean sitting bout duration (min/day)			
	<29.8	29.8-36.4	36.5-43.7	>43.7
<7.0	18*	19	37	53†
7.0-15.5	20	35	39	35
15.6-29.4	47	33	27	23
>29.4	43	42	26	18

*IS, interrupted sitters; †SS, super sitters

Table S2. List of 84 miRs in Human Cardiovascular disease miScript miRNA PCR array

let-7a-5p	let-7b-5p	let-7c	let-7d-5p	let-7e-5p	let-7f-5p
miR-1	miR-100-5p	miR-103a-3p	miR-107	miR-10b-5p	miR-122-5p
miR-124-3p	miR-125a-5p	miR-125b-5p	miR-126-3p	miR-130a-3p	miR-133a
miR-133b	miR-140-5p	miR-142-3p	miR-143-3p	miR-144-3p	miR-145-5p
miR-146a-5p	miR-149-5p	miR-150-5p	miR-155-5p	miR-15b-5p	miR-16-5p
miR-17-5p	miR-181a-5p	miR-181b-5p	miR-182-5p	miR-183-5p	miR-185-5p
miR-18b-5p	miR-195-5p	miR-199a-5p	miR-206	miR-208a	miR-208b
miR-21-5p	miR-210	miR-214-3p	miR-22-3p	miR-221-3p	miR-222-3p
miR-223-3p	miR-224-5p	miR-23a-3p	miR-23b-3p	miR-24-3p	miR-25-3p
miR-26a-5p	miR-26b-5p	miR-27a-3p	miR-27b-3p	miR-29a-3p	miR-29b-3p
miR-29c-3p	miR-302a-3p	miR-302b-3p	miR-30a-5p	miR-30c-5p	miR-30d-5p
miR-30e-5p	miR-31-5p	miR-320a	miR-328	miR-342-3p	miR-365a-3p
miR-378a-3p	miR-423-3p	miR-424-5p	miR-451a	miR-486-5p	miR-494
miR-499a-5p	miR-7-5p	miR-92a-3p	miR-93-5p	miR-98-5p	miR-99a-5p

Table S3. MiRs from the CVD array analysis (N=74) not included in the Top 10 ranking of miRs differentially expressed between IS and SS (Table 2).

	Mean Difference in Relative Expr	Relative Expr, SD	Standardized ATE Rank	standardized ATE	SD Rank
miR-221-3p	9.04	5.46	11	4.16	56
miR-125b-5p	2.06	1.26	12	4.11	23
miR-224-5p	1.97	1.00	13	4.11	19
miR-22-3p	4.84	2.25	14	4.09	41
miR-93-5p	5.93	2.11	15	4.01	37
miR-7-5p	3.85	2.02	16	3.99	35
miR-214-3p	5.54	2.30	17	3.99	43
miR-149-5p	2.07	1.27	18	3.93	24
miR-100-5p	1.26	0.71	19	3.93	12
miR-210	6.21	3.07	20	3.89	47
miR-10b-5p	3.77	2.16	21	3.86	38
miR-1	0.44	0.35	22	3.85	7
miR-107	1.30	0.72	23	3.85	13
miR-181a-5p	1.88	0.89	24	3.79	16
miR-29c-3p	3.35	1.64	25	3.71	29
miR-133a	1.49	1.17	26	3.64	21
miR-18b-5p	2.22	0.97	27	3.62	18
miR-499a-5p	0.11	0.10	28	3.58	1
miR-423-3p	13.14	6.69	29	3.57	62
miR-26b-5p	4.85	2.63	30	3.57	44
miR-124-3p	8.71	4.30	31	3.50	50
miR-16-5p	33.14	14.37	32	3.45	77
miR-208a	1.13	0.50	33	3.45	9
let-7c	18.01	6.18	34	3.42	60
miR-30a-5p	4.17	1.51	35	3.34	26
miR-30d-5p	4.31	1.94	36	3.32	31
miR-26a-5p	40.54	18.24	37	3.30	80
miR-144-3p	3.03	1.57	38	3.29	28
miR-31-5p	7.85	3.21	39	3.21	48
miR-99a-5p	0.68	0.44	40	3.19	8
miR-25-3p	28.93	9.92	41	3.13	69
miR-451a	9.37	7.30	42	2.93	65
miR-30e-5p	2.77	1.17	43	2.91	22
miR-92a-3p	94.44	25.34	44	2.86	83
miR-302a-3p	9.38	4.63	45	2.84	52
miR-328	21.42	10.53	46	2.75	71
miR-30c-5p	28.86	10.08	47	2.68	70
miR-27a-3p	25.35	9.26	48	2.64	67

miR-183-5p	4.60	3.35	49	2.61	49
miR-126-3p	22.88	9.54	50	2.58	68
miR-98-5p	0.65	0.33	51	2.57	6
miR-208b	1.73	0.76	52	2.57	14
miR-365a-3p	0.30	0.18	53	2.55	3
miR-185-5p	3.37	1.97	54	2.47	33
miR-206	10.94	6.37	55	2.43	61
miR-24-3p	2.15	2.06	56	2.42	36
miR-122-5p	0.29	0.14	57	2.40	2
miR-23b-3p	0.92	0.71	58	2.33	11
miR-494	16.53	6.75	59	2.31	63
let-7b-5p	25.47	11.95	60	2.31	73
miR-15b-5p	2.85	2.66	61	2.31	45
let-7f-5p	1.18	0.95	62	2.30	17
miR-21-5p	8.04	7.30	63	2.28	66
miR-130-3p	32.62	12.03	64	2.26	74
miR-27b-3p	4.70	2.16	65	2.21	39
miR-195-5p	31.29	11.05	66	2.13	72
miR-320a	74.51	33.29	67	2.12	84
miR-223-3p	23.16	25.18	68	2.11	82
miR-29b-3p	5.38	2.69	69	2.10	46
miR-29a-3p	11.51	4.82	70	1.98	53
miR-17-5p	15.70	5.87	71	1.98	59
miR-182-5p	22.11	12.76	72	1.97	76
miR-302b-3p	4.62	1.75	73	1.88	30
let-7a-5p	0.84	1.12	74	1.84	20
miR-486-5p	68.52	18.75	75	1.84	81
miR-222-3p	16.40	7.24	76	1.68	64
miR-378a-3p	13.36	5.34	77	1.66	55
miR-150-5p	14.19	5.50	78	1.61	57
miR-342-3p	58.65	17.14	79	1.60	79
miR-143-3p	5.88	1.98	80	1.40	34
miR-125a-5p	16.62	5.85	81	1.37	58
miR-23a-3p	3.66	4.48	82	1.35	51
miR-145-5p	44.65	12.27	83	1.28	75
miR-181b-5p	37.27	15.11	84	0.49	78

List was sorted by standardized ATE and SD of miR levels from IS and SS groups. ATE, average treatment effect: weighted mean difference of miR levels between SS and IS; Standardized ATE, ATE divided by bootstrapped SD of ATE. SD, standard deviation; Expr: expression.

Table S4. Identification of Interrupted Sitter (IS+) and Super Sitter (SS+) groups with augmented representation of Hispanic ethnicity.

MVPA (min/day)	Mean Sitting bout duration (min/day)			
	<29.8	29.8-36.4	36.5-43.7	>43.7
<7.0	18*	19	37	53†
7.0-15.5	13+7*	35	39	28+7†
15.6-29.4	47	33	27	23
>29.4	43	42	26	18

All women who self-identified as Hispanic and who were in the 2nd-lowest quartile of MVPA and 1st and 4th quartiles of mean sitting bout duration (N=7 each) were included to create the amended groups IS+ (n=25) and SS+ (n=60), respectively, with augmented representation of Hispanic ethnicity in the groups.

*IS+, Interrupted Sitters with augmented diversity in Hispanic ethnicity.

†SS+, Super Sitters with augmented diversity in Hispanic ethnicity.

Table S5. Age and Activity-Related Characteristics of IS+ and SS+ Groups

	Total (n=85)	IS+ (n=25)	SS+ (n=60)	*P*-value
Age, mean (sd)	65.7 (6.4)	63.7 (4.7)	66.5 (6.8)	0.06
Activity-related measures, mean (sd)				
Total sitting time; min/day	594.0 (122.7)	440.3(69.9)	658.1 (72.5)	<0.01*
Mean sitting bout duration; min/day	52.0 (26.0)	25.9 (2.8)	62.8 (23.4)	<0.01*
Moderate-to-vigorous activity; min/day	4.8 (3.4)	6.5 (3.8)	4.1 (3.0)	<0.01*
Walking time; min/day	34.9 (29.1)	56.3 (36.9)	26.0 (19.3)	<0.01*

P-values computed using t-tests

*$P<0.05$

Table S6. Correlation analysis among 3 targeted microRNAs

		Let-7d-5p	miR-133b	miR-142-3p
Let-7d-5p	Pearson Correlation	1	0.16	0.426*
	P-value		0.145	0.01
miR-133b	Pearson Correlation	0.16	1	0.16
	P-value	0.145		0.143
miR-142-3p	Pearson Correlation	0.426*	0.16	1
	P-value	0.01	0.143	
*Significant correlation (2-tailed)				

Table S7. Functional pathway analysis of literature-based miR target genes (continuation of Table 4)

KEGG Pathway**	Proteoglycans in cancer	Endocytosis	Protein processing in endoplasmic reticulum	Chronic myeloid leukemia	Hepatitis B	Bacterial invasion of epithelial cells	Thyroid hormone signaling pathway
P-value	2.30E-04	3.42E-03	6.42E-03	7.74E-03	1.86E-02	2.16E-02	2.24E-02
MicroRNAs associated with prolonged sitting	let-7d-5p<br>miR-142-3p	let-7d-5p	let-7d-5p<br>miR-142-3p	miR-142-3p<br>let-7d-5p	let-7d-5p	miR-142-3p	let-7d-5p
Literature-supported target genes (direct target)	*ACTB*<br>*ACTG1*<br>*ARHGEF1*<br>*BRAF*<br>*CASP3*<br>*CCND1*<br>*CDKN1A*<br>*ESR1*<br>*FZD1*<br>*FZD3*<br>*HIF1A*<br>*IGF1R*<br>*ITGAV*<br>*MTOR*<br>*MYC*<br>*NRAS*<br>*PLCG1*<br>*PRKACA*<br>*PTPN6*<br>*RAC1*<br>*RPS6KB2*<br>*SMAD2*<br>*TLR4*<br>*TP53*	*CCR5*<br>*CLTA*<br>*CXCR4*<br>*HSPA8*<br>*IGF1R*<br>*PARD6B*<br>*SMAD2*<br>*SMAD3*<br>*SMAD7*<br>*TGFBR1*<br>*TGFBR2*	*ATF6B*<br>*CAPN1*<br>*ERO1L*<br>*HSPA8*<br>*HSP90AA1*<br>*MAN1A1*<br>*MAPK8*<br>*MOGS*<br>*RPN2*<br>*STT3B*	*AKT2*<br>*BRAF*<br>*CCND1*<br>*CDK4*<br>*CDKN1A*<br>*CRK*<br>*E2F2*<br>*E2F3*<br>*MAPK1*<br>*MYC*<br>*NRAS*<br>*PIK3CD*<br>*PIK3R2*<br>*PIK3R5*<br>*RUNX1*<br>*SMAD3*<br>*SMAD4*<br>*TGFBR1*†<br>*TGFBR2*<br>*TP53*<br>*TGFBR2*	*APAF1*<br>*ATF6B*<br>*CASP3*<br>*CCNA2*<br>*CCND1*<br>*CCNE2*<br>*CDK4*<br>*CDKN1A*<br>*CREBBP*<br>*DDX3X*<br>*DDB1*<br>*E2F2*<br>*E2F3*<br>*FADD*<br>*MAPK1*<br>*MAPK8*<br>*MYC*<br>*NRAS*<br>*TGFBR1*<br>*TLR4*<br>*TP53*<br>*SMAD3*<br>*JAK1*	*CLTA*<br>*CRK*<br>*CTNNB1*<br>*PIK3CD*<br>*PIK3R2*<br>*PIK3R5*<br>*RAC1*	*ACTB*<br>*ACTG1*<br>*CCND1*<br>*CREBBP*<br>*ESR1*<br>*HIF1A*<br>*ITGAV*<br>*MAPK1*<br>*MDM2*<br>*MTOR*<br>*MYC*<br>*NRAS*<br>*PLCG1*<br>*PRKACA*<br>*TP53*

Literature-supported target genes (non-direct target)	*CTSL*	*ADRB2*	*CKAP4*	*CDKN1B*	*MAP2K4*	*ARPC1B*	*ATP1A1*
	FGFR1	*AGAP3*	*DERL1*	*MDM2*†	*MAP3K1*	*CTTN*	*MED13L*
	FLNC	*AP2A1*	*DNAJA1*	*PTPN11*	*PCNA*	*GAB1*	*MED13*
	FZD7	*AP2B1*	*DNAJA2*			*HCLS1*	*NCOA3*
	IGF2	*ARF1*	*DNAJB1*			*WASL*	*PFKP*
	ITPR3	*ARF3*	*DNAJB11*				*SLC16A10*
	MAPK1	*ARF6*	*EDEM2*				*THRA*
	MDM2†	*ASAP1*	*EDEM3*				
	MSN	*EEA1*	*EIF2AK2*				
	PPP1CA	*EHD4*	*EIF2S1*				
	PPP1R12A	*EPN2*	*ERP29*				
	RDX	*LDLR*	*HERPUD1*				
	SDC4	*MDM2*	*LMAN1*				
	TFAP4	*NEDD4*	*MAN1A2*				
	TWIST2	*NEDD4L*	*MAP2K7*				
	VAV2	*PIP5K1C*	*MBTPS1*				
	WNT9A	*RAB11A*	*NPLOC4*				
		RAB22A	*PPP1R15A*				
		RAB5B	*RPN1*				
		RAB5C	*SEC24B*				
		SNF8	*SEC24C*				
		TSG101	*SSR1*				
		VPS25	*SVIP*				
		VPS37C	*TRAM1*				
		ZFYVE20	*UBE2D4*				
			UBE2J1				
			UBE4B				
			XBP1				
			YOD1				
			LMAN1				

**KEGG pathway analysis based on Tarbase v7.0

†Target genes for 2 miRs

Table S8. Functional pathway analysis of literature-based miR target genes (continuation of Table VII)

KEGG Pathway**	Apoptosis	Pancreatic cancer	Colorectal cancer	Estrogen signaling pathway	Prostate cancer	N-Glycan biosynthesis
P-value	2.27E-02	2.61E-02	3.28E-02	3.36E-02	4.65E-02	4.79E-02
MicroRNAs associated with prolonged sitting	let-7d-5p	miR-142-3p	let-7d-5p	miR-142-3p	let-7d-5p	let-7d-5p
Literature-supported target genes (direct target)	*APAF1*<br>*BIRC2*<br>*CAPN1*<br>*CASP3*<br>*FADD*<br>*PRKACA*<br>*TP53*	*AKT2*<br>*E2F2*<br>*PIK3R2*<br>*PIK3R5*<br>*PIK3CD*<br>*RAC1*<br>*SMAD4*<br>*TGFBR1*	*BRAF*<br>*CASP3*<br>*CCND1*<br>*MAPK1*<br>*MAPK8*<br>*MSH6*<br>*MYC*<br>*RAC1*<br>*SMAD2*<br>*SMAD3*<br>*TGFBR1*<br>*TGFBR2*<br>*TP53*	*AKT2*<br>*PIK3CD*<br>*PIK3R2*<br>*PIK3R5*	*AR*<br>*BRAF*<br>*CCND1*<br>*CCNE2*<br>*CDKN1A*<br>*CREBBP*<br>*E2F2*<br>*E2F3*<br>*FGFR1*<br>*HSP90AA1*<br>*IGF1R*<br>*MAPK1*<br>*MDM2*<br>*MTOR*<br>*NRAS*<br>*TP53*	*ALG3*<br>*ALG8*<br>*DOLK*<br>*DPM2*<br>*MAN1A1*<br>*MGAT1*<br>*MGAT4A*<br>*MOGS*<br>*RPN1*<br>*RPN2*<br>*STT3B*<br>*ST6GAL1*
Literature-supported target genes (non-direct target)	*BIRC3*<br>*DFFA*<br>*IL1R1*<br>*PRKAR1B*<br>*PRKAR2A*<br>*TNFRSF10B*<br>*XIAP*			*ATF2*<br>*CREB3L2*<br>*CREB5*<br>*GNAQ*<br>*GNAS*<br>*HSPA1B*<br>*PRKCD*	*PDGFB*	

**KEGG pathway analysis based on Tarbase v7.0.

†Target genes for 2 miRs.

Table S9. Primer assays for 5 selected miRs and control panels used for validation studies

miScript Primer Assay	Targets mature miRs	Target organism	Vendor	Catalog#
Hs_miR-199a_1	miR-199a-5p	Homo sapiens	Qiagen	MS00006741
Hs_le7d_1	let-7d-5p	Homo sapiens	Qiagen	MS00003136
Hs_miR-140_1	miR-140-5p	Homo sapiens	Qiagen	MS00003500
Hs_miR-142-3p_2	miR-142-3p	Homo sapiens	Qiagen	MS00031451
Hs_miR-133b_2	miR-133b	Homo sapiens	Qiagen	MS00031430
Hs_SNORD61_11	SNORD61 small RNA	Homo sapiens	Qiagen	MS00033705
Cel-miR-39_1	cel-miR-39-3p	C-elegans	Qiagen	MS00019789

Table S10. Target genes of the 3 miRs

**let-7d-5p**	**miR-133b**	**miR-142-3p**
A1CF	*AAK1*	*ABCA9*
AAK1	*ABCC1*	*ABCD3*
AASS	*ABCD2*	*ABI2*
ABCB9	*ABCG4*	*ABL2*
ABCC10	*ABL2*	*AC012123.1*
ABCC5	*ACAT2*	*ACBD5*
ABHD17C	*ACTR1A*	*ACVR2A*
ABL2	*ADAMTS5*	*ADAMTS3*
AC008443.1	*ADARB2*	*ADCY9*
AC010441.1	*ADCY5*	*AFF1*
AC023632.1	*ADCY6*	*AFF2*
AC068987.1	*ADCYAP1*	*AKIRIN2*
AC140061.12	*ADRA2B*	*AKT1S1*
ACAP3	*AFAP1*	*ANK3*
ACER2	*AFTPH*	*ANKFY1*
ACER3	*AGRP*	*ANKRD11*
ACPP	*AIF1L*	*ANKRD33B*
ACSL6	*AK2*	*ANKRD40*
ACVR1B	*AKAP9*	*ANKS1A*
ACVR1C	*AL117190.3*	*AP5M1*
ACVR2A	*ALDH5A1*	*APC*
ACVR2B	*AMD1*	*ARHGAP12*
ADAM15	*ANAPC1*	*ARHGEF12*
ADAMTS1	*ANK2*	*ARHGEF2*
ADAMTS14	*ANKRD12*	*ARID5B*
ADAMTS15	*ANKRD28*	*ARL1*
ADAMTS5	*ANKRD44*	*ARL15*
ADAMTS8	*ANKRD46*	*ARNTL*
ADCY9	*ANKRD52*	*ARRDC3*
ADIPOR2	*AOX1*	*ASAP2*
ADRB1	*AP1AR*	*ASB7*
ADRB2	*AP1B1*	*ASH1L*
ADRB3	*AP2M1*	*ATF7IP*
ADRBK2	*AP4E1*	*ATG16L1*
AEN	*AQP1*	*ATP1B1*
AFF2	*ARFIP2*	*ATP2A2*
AGAP1	*ARHGAP12*	*ATXN1L*
AGO1	*ARHGAP24*	*ATXN2*
AGO3	*ARHGAP39*	*ATXN7*
AGO4	*ARHGAP9*	*BACH1*
AGPAT6	*ARHGDIA*	*BACH2*
AGPAT9	*ARL3*	*BAZ1A*
AHCTF1	*ARNTL2*	*BCL2L1*
AIFM1	*ARPC5*	*BCLAF1*
AKAP5	*ARRB1*	*BIRC3*
AKAP6	*ARX*	*BNC2*
AKT2	*ASB1*	*BOD1*

ALDH6A1	*ASH1L*	*BRWD3*
ALKBH1	*ATOX1*	*BTBD7*
ALPK3	*ATP2A2*	*BTLA*
AMER3	*ATP6AP2*	*C16orf70*
AMMECR1L	*ATRX*	*C18orf25*
AMOT	*B3GALNT1*	*C20orf112*
AMT	*B3GNT2*	*C20orf194*
ANGPTL2	*B3GNT9*	*C4orf32*
ANKFY1	*BAMBI*	*C5orf24*
ANKRD28	*BAZ2A*	*C6orf47*
ANKRD33B	*BCL2L1*	*C9orf72*
ANKRD46	*BCL2L2*	*CA5B*
ANKRD52	*BCORL1*	*CASK*
AP1S1	*BHLHE41*	*CASZ1*
APBA1	*BICC1*	*CCDC6*
APBB3	*BNC2*	*CCNJ*
APC2	*BNIP3L*	*CCNT2*
APPBP2	*BTBD10*	*CD84*
ARG2	*BTBD3*	*CDC14A*
ARHGAP20	*C11orf58*	*CELF2*
ARHGAP28	*C12orf43*	*CFL2*
ARHGEF15	*C17orf96*	*CHMP3*
ARHGEF38	*C1QL1*	*CHRNE*
ARHGEF7	*C20orf194*	*CHURC1-FNTB*
ARID3A	*C2orf69*	*CIITA*
ARID3B	*CACNA1B*	*CLCN5*
ARL4D	*CACUL1*	*CLIC4*
ARL5A	*CALM1*	*CLOCK*
ARL5B	*CAMK1D*	*CLTA*
ARL6IP6	*CAP1*	*CNIH4*
ARMC8	*CAPN15*	*COG4*
ARPP19	*CAPN5*	*COL24A1*
ARRDC4	*CASC3*	*COPG1*
ASAP1	*CCDC117*	*COPS7A*
ATAD2B	*CCDC144A*	*CPEB2*
ATG16L1	*CCDC176*	*CRK*
ATG4B	*CCDC30*	*CRTAM*
ATP2A2	*CCNDBP1*	*CTNND1*
ATP2B3	*CCNT1*	*CTTN*
ATP2B4	*CD47*	*CXADR*
ATP6V1C1	*CDCA8*	*CYSLTR2*
ATP8B4	*CDIP1*	*DCUN1D4*
ATPAF1	*CDK13*	*DHX30*
ATXN1L	*CDK5R1*	*DIDO1*
ATXN7L3	*CDK8*	*DIRC2*
ATXN7L3B	*CDYL2*	*DYNC1LI2*
B3GALT1	*CECR6*	*EDEM3*
B3GAT1	*CELF1*	*EHF*
B3GAT3	*CELF5*	*EML4*

B3GNT1	*CELF6*	*EPN1*
B3GNT7	*CEP128*	*ERC1*
BACE2	*CEP85L*	*ERG*
BACH1	*CERS2*	*FAM114A1*
BAHD1	*CETN3*	*FAM127A*
BBX	*CGGBP1*	*FAM127B*
BCAP29	*CHD2*	*FAM208B*
BCAT1	*CHD3*	*FAM73A*
BCL2L1	*CHRM3*	*FBXO21*
BCL7A	*CKAP4*	*FBXO3*
BDP1	*CLCN6*	*FBXO45*
BEGAIN	*CLTA*	*FKBP1A*
BEND4	*CMPK1*	*FLVCR1*
BIN3	*CMTM6*	*FMNL2*
BLOC1S5	*CNKSR2*	*FNBP1L*
BLOC1S6	*CNN2*	*FNDC3A*
BMP2	*CNNM2*	*FNTB*
BMP5	*CNNM4*	*FOXO1*
BMPR1A	*CNOT1*	*FOXO4*
BNC2	*CNTNAP1*	*GAB1*
BRWD3	*COL1A1*	*GFI1*
BSN	*COL25A1*	*GHR*
BSND	*COL8A1*	*GNAQ*
BTBD3	*CORO1C*	*GNAS*
BTBD9	*CPLX2*	*GNB2*
BTF3L4	*CPNE3*	*GOLGA1*
BTG2	*CREB5*	*GPCPD1*
BZW1	*CRK*	*GPR137C*
BZW2	*CRTAM*	*GPRIN3*
C11orf57	*CSNK1G3*	*GTF2A1*
C11orf84	*CSRNP1*	*HAVCR1*
C14orf28	*CSTF2*	*HEATR5A*
C15orf39	*CTBP2*	*HECTD1*
C15orf41	*CTGF*	*HHIP*
C16orf52	*CTSV*	*HIPK1*
C17orf85	*CUL4B*	*HMGA1*
C19orf26	*CUX2*	*HMGA2*
C19orf47	*CYLD*	*HMGB1*
C1GALT1	*DAPK2*	*HNRNPC*
C1orf147	*DBNL*	*HSPE1-MOB4*
C20orf112	*DCBLD1*	*IER3*
C20orf194	*DCLRE1A*	*IL1RAPL1*
C2orf88	*DCP1A*	*IL6ST*
C3orf52	*DCUN1D4*	*IL7R*
C5orf51	*DDX19B*	*INHBA*
C6orf203	*DDX3X*	*INPP5A*
C6orf211	*DDX3Y*	*IPMK*
C8orf58	*DESI1*	*IPO7*
C9orf40	*DIAPH2*	*IRAK1*

C9orf41	*DLG2*	*ITGA8*
CACFD1	*DLGAP3*	*ITGAV*
CACNA1E	*DMXL1*	*ITGB8*
CACNA1I	*DNAJB1*	*ITPKB*
CACNB4	*DNAJC3*	*ITPR3*
CACNG4	*DOLPP1*	*JMJD1C*
CADM2	*DOT1L*	*JMJD7-PLA2G4B*
CALM1	*DSN1*	*KAT2B*
CALN1	*DSTYK*	*KAT7*
CALU	*DUSP1*	*KCNJ6*
CANT1	*DUSP7*	*KCNS2*
CAPN3	*DYNC1LI2*	*KDELR2*
CASK	*E2F7*	*KDM6A*
CASKIN1	*EDEM1*	*KIF5B*
CASP3	*EEF1A1*	*KLF13*
CASP5	*EFNA4*	*KMT2A*
CBFA2T3	*EGFR*	*LCOR*
CBL	*EIF3J*	*LLGL2*
CBX2	*EIF4A1*	*LMO3*
CBX5	*ELAVL1*	*LPP*
CCDC141	*ELF2*	*LRP1B*
CCDC144A	*ELF3*	*LRRC1*
CCDC71L	*ELFN1*	*LRRC17*
CCDC93	*ELMO1*	*LRRC32*
CCL3	*ELP5*	*LRRC59*
CCL3L1	*EMP2*	*MANBAL*
CCL3L3	*ENC1*	*MAP2*
CCL7	*ENPEP*	*MAP2K6*
CCND1	*ENPP5*	*MAP3K11*
CCND2	*ENPP6*	*MAP4K3*
CCNF	*ENTPD7*	*MARCH1*
CCNJ	*EPHA7*	*MARCKS*
CCNJL	*EPHB4*	*MARK3*
CCNT2	*EPS15*	*MBD6*
CCNY	*ERMP1*	*MEF2C*
CCR7	*ESRRG*	*MEIS1*
CCSAP	*ESYT1*	*MGAT4A*
CD200R1	*ESYT2*	*MLXIP*
CD86	*ETF1*	*MMGT1*
CDC25A	*EXD2*	*MOB4*
CDC34	*EYA1*	*MOCS1*
CDC42SE1	*EYA4*	*MORF4L1*
CDCA8	*FAF2*	*MORF4L2*
CDH22	*FAM102B*	*MRFAP1*
CDK14	*FAM117B*	*MSI2*
CDK6	*FAM13A*	*MTCH1*
CDK8	*FAM160B1*	*MTMR9*
CDKN1A	*FAM193B*	*MYH10*
CDV3	*FAM199X*	*MYH9*

CDYL	*FAM19A5*	*MYLK*
CEBPD	*FAM208A*	*MYO5A*
CECR2	*FAM43B*	*NCK2*
CECR6	*FAM46A*	*NDC1*
CELF1	*FAM46C*	*NFIB*
CEP135	*FAM49A*	*NHSL1*
CEP164	*FAM57A*	*NKX2-3*
CEP85L	*FAM73A*	*NOL7*
CERCAM	*FARP1*	*NR1D2*
CFL2	*FBN1*	*NR2F6*
CGNL1	*FBN2*	*NR3C1*
CHD4	*FBXL19*	*NSF*
CHD7	*FBXL2*	*NSUN3*
CHD9	*FBXW11*	*NUCKS1*
CHIC1	*FGF1*	*P2RY1*
CHRD	*FGFR1*	*PAFAH1B2*
CHRFAM7A	*FLVCR1*	*PAN3*
CHRNA7	*FOSL2*	*PAPD7*
CHST3	*FOXC1*	*PARD6B*
CHSY3	*FOXG1*	*PARP8*
CHUK	*FOXK2*	*PATZ1*
CLASP2	*FOXL2*	*PCGF3*
CLCN5	*FOXN3*	*PDE4B*
CLDN12	*FOXP1*	*PDE8A*
CLOCK	*FOXP2*	*PGRMC2*
CLP1	*FOXP4*	*PHF12*
CLPB	*FOXQ1*	*PICALM*
CMTM6	*FRS2*	*PIK3CG*
CNNM2	*FSCN1*	*PIK3R6*
CNOT2	*FST*	*PLCB1*
CNOT6L	*FTL*	*PLEKHA3*
CNTRL	*FURIN*	*PMAIP1*
COIL	*GABARAPL1*	*POMGNT1*
COL11A1	*GABBR2*	*PPP1R10*
COL14A1	*GABPB2*	*PPP1R15B*
COL15A1	*GABRB1*	*PPP1R37*
COL1A1	*GALNT16*	*PPP3CA*
COL1A2	*GARNL3*	*PPP3R1*
COL24A1	*GBA2*	*PRLR*
COL27A1	*GCH1*	*PROM1*
COL3A1	*GCLC*	*PROX1*
COL4A1	*GDF11*	*PRRG1*
COL4A2	*GDI1*	*PSMB5*
COL4A3BP	*GDI2*	*PSMD11*
COL4A5	*GDNF*	*PTBP3*
COL4A6	*GID8*	*PTPN23*
COL5A2	*GIGYF1*	*PUM1*
COL9A1	*GLRA2*	*PURB*
COL9A3	*GMEB1*	*QKI*

CPA4	*GNB4*	*RAB11A*
CPD	*GPM6A*	*RAB11FIP2*
CPEB1	*GPR173*	*RAB12*
CPEB2	*GRIA2*	*RAB1A*
CPEB3	*GRID2*	*RAB2A*
CPEB4	*GRM5*	*RAB3A*
CPED1	*GRM7*	*RAB3D*
CPM	*GXYLT1*	*RAB40C*
CPSF4	*GZF1*	*RAC1*
CRB2	*HAPLN1*	*RAP1GAP2*
CRBN	*HECW1*	*RARG*
CRCT1	*HHIP*	*RASSF3*
CRK	*HIC2*	*RBFOX2*
CRTAP	*HIVEP2*	*RBM27*
CRY2	*HLF*	*RBM47*
CSNK2A1	*HLTF*	*RBMS1*
CSRNP3	*HMGXB3*	*RERE*
CTB-54O9.9	*HOXA9*	*RFWD3*
CTDSPL2	*HOXB5*	*RGL2*
CTIF	*HOXD1*	*RHEB*
CTNS	*HS2ST1*	*RHOBTB3*
CTPS1	*HS3ST5*	*RICTOR*
CXorf36	*HSPA13*	*RIMKLB*
CYB561D1	*HSPA4L*	*RLF*
CYP19A1	*ICK*	*RNF103-CHMP3*
CYP46A1	*ID4*	*RNF157*
CYTH3	*IDH1*	*RNF24*
DAB1	*IFIT2*	*RNF38*
DAGLA	*IGF1R*	*ROCK2*
DAPK1	*IL6ST*	*RPRD1A*
DARS2	*IMPG1*	*RREB1*
DCAF15	*INSR*	*RSF1*
DCAF8	*IQGAP2*	*S1PR3*
DCUN1D2	*ISCA2*	*SEC22A*
DCUN1D3	*ITPRIPL2*	*SEMA5B*
DCX	*JAZF1*	*SERAC1*
DDI2	*JDP2*	*SERP1*
DDN	*KCNA6*	*SETD1B*
DDX19A	*KCND3*	*SGK1*
DDX19B	*KCNJ12*	*SH2B1*
DDX26B	*KCNK7*	*SH3GLB1*
DENND6A	*KCTD15*	*SIK1*
DHX57	*KCTD16*	*SIK2*
DIAPH2	*KCTD20*	*SKP1*
DICER1	*KDM2A*	*SLC17A5*
DKK3	*KIAA0141*	*SLC1A3*
DLC1	*KIAA0232*	*SLC25A22*
DLGAP4	*KIAA0430*	*SLC35A1*
DLST	*KIAA1024*	*SLC35F5*

DMD	*KIAA1045*	*SLC37A3*
DMP1	*KIAA1239*	*SLC38A4*
DNA2	*KIAA1429*	*SLC6A11*
DNAH10OS	*KIAA1432*	*SLC7A11*
DNAJA2	*KIAA1522*	*SLCO4C1*
DNAJB14	*KIAA1549*	*SMG1*
DNAJB9	*KIF21A*	*SMUG1*
DNAJC1	*KIF3C*	*SNX18*
DNAJC10	*KIRREL*	*SOCS6*
DNAL1	*KLF7*	*SOX11*
DOCK3	*KLHDC10*	*SOX5*
DPF2	*KLHL11*	*SP4*
DPH1	*KLHL2*	*SP8*
DPH3	*KLHL42*	*SPNS1*
DPP3	*KLHL9*	*SPRED1*
DPYSL3	*KMT2C*	*SS18*
DSG3	*KPNA6*	*STAM*
DST	*LAMB3*	*STAM2*
DTX2	*LANCL2*	*STAU1*
DTX4	*LASP1*	*STRN3*
DUSP1	*LDLRAP1*	*STX12*
DUSP16	*LDOC1*	*STX7*
DUSP22	*LEPROTL1*	*SUCO*
DUSP4	*LETMD1*	*SYPL1*
DUSP7	*LGALS8*	*TAB2*
DUSP9	*LHFP*	*TAGAP*
DVL3	*LHX5*	*TAOK1*
DYRK1A	*LHX9*	*TARDBP*
DYRK2	*LIN28B*	*TBC1D2B*
DZIP1	*LIN7A*	*TBL1X*
E2F2	*LIN7C*	*TBL1XR1*
E2F5	*LOXL4*	*TCEB3*
E2F6	*LPGAT1*	*TEAD1*
EDA	*LPHN1*	*TESK2*
EDEM3	*LPIN2*	*TET3*
EDN1	*LRRC2*	*TEX2*
EEA1	*LRRC7*	*TFG*
EEF2K	*LTBP1*	*TGFB2*
EFHD2	*LUZP1*	*TGFBR1*
EGLN2	*LZTS2*	*THSD4*
EGR3	*LZTS3*	*TIPARP*
EIF2B2	*MAEA*	*TIRAP*
EIF2S2	*MAFG*	*TMED7*
EIF4G2	*MAML1*	*TMEFF2*
EIF5B	*MAML3*	*TMEM110*
ELF4	*MAN1A2*	*TMEM115*
ELK4	*MAP3K2*	*TMEM200B*
ELOVL4	*MAP3K3*	*TMEM55B*
EMB	*MAP7*	*TMEM59*

ENTPD7	*MCL1*	*TNKS*
EOGT	*MECOM*	*TNRC18*
EPHA3	*MECP2*	*TP53INP2*
EPHA4	*MED12L*	*TPM4*
ERCC4	*MED8*	*TSEN34*
ERCC6	*MEIS1*	*TTBK2*
ERGIC1	*MEIS2*	*TWF1*
ERGIC2	*METTL21B*	*TXLNA*
ERO1L	*MFSD6*	*UNKL*
ERP29	*MID2*	*USP6NL*
ESPL1	*MIER3*	*UTRN*
ETNK1	*MKL2*	*UTY*
ETNK2	*MLLT3*	*VAMP3*
ETV3	*MLXIP*	*VANGL1*
EXOC5	*MMGT1*	*WASL*
EZH1	*MMP14*	*WHSC1L1*
FADS3	*MMP15*	*WIZ*
FAM103A1	*MPPED1*	*WNK3*
FAM104A	*MRC2*	*XIAP*
FAM118A	*MRFAP1*	*XPO1*
FAM135A	*MRGBP*	*ZBTB10*
FAM155A	*MRPL44*	*ZBTB20*
FAM160B2	*MSN*	*ZBTB41*
FAM178A	*MTMR4*	*ZCCHC14*
FAM189A1	*MTX3*	*ZCCHC24*
FAM208A	*MYH9*	*ZEB1*
FAM214B	*MYO9B*	*ZEB2*
FAM222B	*MYRF*	*ZFP36L2*
FAM43A	*NAA40*	*ZFP91*
FAM83G	*NAGS*	*ZFR*
FAM84B	*NCAM2*	*ZMYND8*
FARP1	*NCAN*	*ZNF217*
FAS	*NCKAP5L*	*ZNF395*
FASLG	*NDRG1*	*ZNF618*
FASTK	*NEBL*	*ZNF644*
FAXC	*NELFA*	*ZNF652*
FBXL19	*NELL2*	*ZNF827*
FBXO21	*NFAT5*	*ZNF831*
FBXO30	*NFIA*	*ZZZ3*
FBXO32	*NFIB*	
FBXO45	*NFYA*	
FGD6	*NIPA1*	
FGF11	*NIPA2*	
FGF5	*NKD1*	
FIGN	*NLGN2*	
FKRP	*NMT1*	
FNDC3A	*NR6A1*	
FNDC3B	*NRG3*	
FNIP1	*NRIP3*	

FNIP2	*NTNG1*
FOPNL	*NUP153*
FOXP1	*NUP160*
FOXP2	*NUS1*
FRAS1	*ORAI2*
FREM2	*ORC4*
FRMD4B	*OTUD4*
FRMD5	*OTUD7B*
FRS2	*PAN3*
FSD2	*PANK3*
FSTL4	*PAOX*
FZD3	*PAPD5*
FZD4	*PARD6G*
G3BP1	*PARVA*
GAB2	*PAWR*
GABBR2	*PAX7*
GABPA	*PCDH1*
GABRA6	*PCDH17*
GABRB1	*PCSK5*
GALC	*PCSK6*
GALE	*PDE10A*
GALNT1	*PDE1C*
GALNT15	*PDE7A*
GALNT2	*PDE8B*
GALNT4	*PDIK1L*
GAN	*PDK3*
GAREM	*PEAK1*
GAS7	*PEX5L*
GATM	*PFAS*
GCNT4	*PFKFB3*
GDAP2	*PFN2*
GDF6	*PHTF2*
GDPD1	*PIK3C2A*
GEMIN7	*PIKFYVE*
GHR	*PIP4K2B*
GIPC1	*PITPNB*
GJA9	*PITPNM2*
GJC1	*PITPNM3*
GK5	*PKNOX1*
GLRX	*PLCL2*
GNAL	*PLEKHA3*
GNG5	*PLEKHA8*
GNPTAB	*PML*
GNS	*POLH*
GOLGA4	*POU4F1*
GOLGA7	*POU6F2*
GOLT1B	*PPFIA2*
GOPC	*PPFIA3*
GPATCH2	*PPM1H*

GPATCH3	*PPP1R9B*
GPC4	*PPP2CA*
GPCPD1	*PPP2CB*
GPR132	*PPP2R2D*
GPR137	*PPP2R4*
GPR137C	*PPP2R5E*
GPR156	*PPP6R3*
GPR157	*PRDM1*
GPR26	*PRDM16*
GPR61	*PRDM6*
GPR63	*PREX1*
GRAMD1B	*PRKAB1*
GREB1	*PRKCB*
GREB1L	*PRMT6*
GRID2IP	*PROX1*
GRIK2	*PRPF38A*
GRIN2B	*PRRT2*
GRIN3A	*PSD3*
GRPEL2	*PSEN1*
GSG1L	*PTBP1*
GTF2I	*PTBP2*
GXYLT1	*PTBP3*
GYG2	*PTMA*
HABP4	*PTPN22*
HAND1	*PTPRD*
HAND2	*PTPRK*
HAS2	*PTPRT*
HBEGF	*PTPRZ1*
HCFC2	*PURB*
HDHD1	*PWWP2B*
HDLBP	*QKI*
HDX	*RAB11FIP4*
HECTD2	*RAB27B*
HECTD4	*RAB30*
HELZ	*RAB5C*
HIC1	*RAD51L3-RFFL*
HIC2	*RAP2C*
HIF1AN	*RAPH1*
HIF3A	*RARB*
HIP1	*RAVER1*
HIPK2	*RB1CC1*
HK2	*RBM23*
HLF	*RBMS1*
HMGA1	*RBMX*
HMGA2	*RBMXL1*
HNRNPA1	*RBPJ*
HOOK1	*RCE1*
HOXA1	*RFC1*
HOXA9	*RFFL*

HOXB1	*RFX3*
HOXC11	*RFX6*
HOXD1	*RHOA*
HS2ST1	*RIMS1*
HSPE1-MOB4	*RLN2*
HTR1E	*RP1-170O19.20*
HTR4	*RPAP2*
ICK	*RPL17-C18orf32*
ICMT	*RRP15*
ICOS	*RTN4RL1*
IDH2	*RUNX1T1*
IGDCC3	*RWDD2A*
IGDCC4	*SACM1L*
IGF1	*SAMD5*
IGF1R	*SCN1A*
IGF2BP1	*SCN2B*
IGF2BP2	*SCOC*
IGF2BP3	*SDC2*
IGLON5	*SEC61B*
IGSF1	*SEPHS2*
IKBKAP	*SESN1*
IKBKE	*SESN3*
IKZF2	*SETD6*
IL10	*SF3B1*
IL13	*SF3B4*
IL6R	*SFMBT2*
IMPG2	*SFT2D3*
INO80D	*SFXN2*
INPP5A	*SFXN5*
INSR	*SGK1*
INTS2	*SGMS2*
INTU	*SGPP1*
IPO11	*SGSM1*
IPO9	*SGTB*
IQCB1	*SH3GL2*
IRGQ	*SH3PXD2B*
IRS2	*SHISA5*
ISLR	*SIMC1*
ITGA1	*SIRT1*
ITGB3	*SIX5*
ITGB8	*SLC25A36*
ITSN1	*SLC25A39*
JOSD1	*SLC25A51*
KATNAL1	*SLC26A2*
KATNBL1	*SLC27A4*
KCMF1	*SLC30A3*
KCNA6	*SLC30A5*
KCNC1	*SLC30A7*
KCNC2	*SLC33A1*

KCNC3	*SLC35F5*
KCNC4	*SLC39A1*
KCNJ11	*SLC41A1*
KCNJ3	*SLC4A1*
KCNJ6	*SLC4A8*
KCNK6	*SLC50A1*
KCNQ4	*SLC6A1*
KCNV1	*SLC6A6*
KCTD10	*SLC7A2*
KCTD15	*SLC7A8*
KCTD16	*SLC8A1*
KCTD17	*SLC8A2*
KCTD21	*SLMO2*
KDM3A	*SMARCA2*
KHNYN	*SMARCD1*
KIAA0391	*SMC2*
KIAA0895L	*SMIM14*
KIAA0930	*SMPD4*
KIAA1147	*SNRK*
KIAA1161	*SNX15*
KIAA1244	*SNX30*
KIAA1467	*SOBP*
KIAA1549	*SOCS2*
KIAA1919	*SOGA1*
KIAA1958	*SOGA3*
KIAA2022	*SORBS3*
KIF21B	*SOX4*
KIF2A	*SP1*
KLF8	*SP3*
KLF9	*SPOPL*
KLHDC8B	*SPRN*
KLHL13	*SPRY4*
KLHL23	*SPTBN1*
KLHL31	*SPTLC2*
KLHL36	*SPTSSA*
KLHL6	*SPTY2D1*
KLK10	*SRGAP3*
KMT2D	*SRP19*
KMT2E	*SRPK1*
KPNA1	*SRSF10*
KPNA4	*ST8SIA3*
KPNA5	*STARD13*
KREMEN1	*STOM*
L2HGDH	*STX5*
LBH	*STXBP6*
LBR	*SUMO1*
LCOR	*SUPT16H*
LCORL	*SV2A*
LDB1	*SVOP*

LEPROTL1	*SYAP1*
LGR4	*SYNPO2L*
LIMD1	*SYNRG*
LIMD2	*SYT1*
LIMK2	*SYT2*
LIN28A	*SYT9*
LIN28B	*TAGLN2*
LINGO1	*TAOK1*
LIPH	*TBL1X*
LIPT2	*TBPL1*
LMLN	*TCF7*
LMX1A	*TEAD1*
LONRF3	*TENM1*
LOR	*TET3*
LOXL4	*TFAP2D*
LPGAT1	*TFE3*
LPHN3	*TFG*
LRFN4	*TG*
LRIG2	*TGFB2*
LRIG3	*TGFBR1*
LRRC10B	*TGOLN2*
LRRC20	*THNSL1*
LRRC59	*THRAP3*
LRRC8B	*THUMPD3*
LSM11	*TIMM17A*
LTN1	*TK2*
LUC7L3	*TM9SF3*
LYVE1	*TMEM110*
LZIC	*TMEM158*
MAP3K1	*TMEM167A*
MAP3K13	*TMEM170B*
MAP3K2	*TMEM180*
MAP3K3	*TMEM200B*
MAP3K9	*TMEM33*
MAP4K3	*TMEM57*
MAP4K4	*TMEM87B*
MAPK1IP1L	*TMOD3*
MAPK6	*TNFRSF10B*
MAPK8	*TNIK*
MARCH9	*TNRC6B*
MARS2	*TOB2*
MBD2	*TOR2A*
MBTD1	*TPD52*
MBTPS2	*TPM4*
MDFI	*TRAF3*
MDM4	*TRAF3IP2*
MED28	*TRAM2*
MED6	*TRHDE*
MED8	*TRIM44*

MEF2C	*TRIM55*
MEF2D	*TRIM56*
MEGF11	*TSPAN18*
MEIS1	*TTC31*
MEIS2	*TTPAL*
MESDC1	*TTYH3*
MEX3A	*TUBB1*
MFAP3L	*TXLNA*
MFSD4	*TYRO3*
MGA	*UBA2*
MGAT4A	*UBE2K*
MGLL	*UBE2Q1*
MIB1	*UBE2Z*
MICALCL	*UBFD1*
MIEF1	*UBXN7*
MIOS	*UCK2*
MLLT10	*UNC13A*
MLXIP	*UNC80*
MMP11	*UNK*
MOB4	*UNKL*
MON2	*USH1G*
MPST	*USP32*
MRS2	*USP38*
MSANTD3	*USP6*
MSI2	*VAMP2*
MSN	*VAMP3*
MTDH	*VAPB*
MTMR12	*VAT1*
MTMR3	*VEGFC*
MTUS1	*VKORC1*
MVB12B	*VPS13B*
MXD1	*VPS4B*
MYCBP	*VPS54*
MYCL	*VTI1B*
MYCN	*WASF2*
MYO1F	*WDR59*
MYO5B	*WDTC1*
MYRIP	*WIPF2*
NAA20	*WIPI2*
NAA30	*WNT4*
NAP1L1	*WTAP*
NAPEPLD	*XPO1*
NAT8L	*XPO4*
NCOA1	*XXbac-BPG32J3.20*
NCOR1	*XXYLT1*
NDST2	*YES1*
NEDD4L	*YIPF4*
NEFM	*YPEL2*
NEK3	*ZBTB10*

NEK9	*ZBTB14*
NGF	*ZBTB16*
NHLRC2	*ZBTB20*
NHLRC3	*ZBTB38*
NID2	*ZBTB4*
NIPA1	*ZBTB7B*
NIPAL4	*ZC3H11A*
NKAP	*ZC3H14*
NKD1	*ZC3H7A*
NKIRAS2	*ZC3HAV1*
NLK	*ZHX1*
NLN	*ZHX3*
NME4	*ZIC3*
NME6	*ZNF131*
NNT	*ZNF217*
NOS1	*ZNF280C*
NOVA1	*ZNF322*
NPEPL1	*ZNF354A*
NR6A1	*ZNF354B*
NRAS	*ZNF362*
NRK	*ZNF385A*
NSD1	*ZNF395*
NSMCE2	*ZNF436*
NTN1	*ZNF664*
NUMBL	*ZNF710*
NUP155	*ZNF740*
NXT2	*ZNRF3*
NYNRIN	
OLR1	
ONECUT1	
ONECUT2	
ONECUT3	
OSBPL3	
OSMR	
OSTF1	
OTOF	
OTUB2	
OTUD3	
P2RX1	
P4HA2	
PACS2	
PAG1	
PAK1	
PALD1	
PALM3	
PANX2	
PAPPA	
PARD6B	
PARM1	

PARP8
PAWR
PAX2
PAX3
PBX1
PBX2
PBX3
PCDH19
PCDH20
PCGF3
PCTP
PCYOX1
PCYT1B
PDE12
PDGFB
PDHB
PDP2
PDPR
PDSS1
PDZD8
PEG10
PEX11B
PGM2L1
PGRMC1
PHACTR2
PHF8
PI4K2B
PIAS4
PIGA
PIGU
PIK3CA
PIK3IP1
PITPNM3
PKIA
PKN2
PLA2G15
PLA2G3
PLAGL2
PLCXD3
PLD3
PLD5
PLEKHA3
PLEKHA8
PLEKHG7
PLEKHH1
PLEKHO1
PLXNA2
PLXNA4
PLXNC1

PLXND1<br>*PMAIP1*<br>*POC1B-GALNT4*<br>*POGLUT1*<br>*POGZ*<br>*POLR2D*<br>*POLR3D*<br>*POTEE*<br>*POTEG*<br>*POTEM*<br>*POU2F1*<br>*POU6F2*<br>*PPAPDC2*<br>*PPARGC1A*<br>*PPARGC1B*<br>*PPP1R12B*<br>*PPP1R15B*<br>*PPP1R16B*<br>*PPP2R2A*<br>*PPP3CA*<br>*PPTC7*<br>*PQLC2*<br>*PRDM1*<br>*PRDM5*<br>*PRKAA2*<br>*PRKAB2*<br>*PRKAR2A*<br>*PRLR*<br>*PRPF38B*<br>*PRR14L*<br>*PRR18*<br>*PRRX1*<br>*PRSS22*<br>*PSENEN*<br>*PSORS1C2*<br>*PTAFR*<br>*PTAR1*<br>*PTPRD*<br>*PTPRU*<br>*PURB*<br>*PXDN*<br>*PXMP4*<br>*PXT1*<br>*PYGO2*<br>*QARS*<br>*RAB11FIP4*<br>*RAB15*<br>*RAB22A*<br>*RAB30*	

RAB3GAP2<br>*RAB40C*<br>*RAB8B*<br>*RADIL*<br>*RAG1*<br>*RALB*<br>*RALGPS1*<br>*RANBP2*<br>*RASGRP1*<br>*RASL10A*<br>*RASL10B*<br>*RAVER2*<br>*RB1*<br>*RBFOX1*<br>*RBFOX2*<br>*RBM38*<br>*RBMS1*<br>*RBMS2*<br>*RBPJ*<br>*RCN1*<br>*RCSD1*<br>*RDH10*<br>*RDX*<br>*REEP1*<br>*REL*<br>*RFX6*<br>*RGAG1*<br>*RGMA*<br>*RGS16*<br>*RGS17*<br>*RICTOR*<br>*RIMKLA*<br>*RIMS3*<br>*RIOK3*<br>*RNF152*<br>*RNF165*<br>*RNF170*<br>*RNF20*<br>*RNF217*<br>*RNF38*<br>*RNF44*<br>*RNF7*<br>*RNFT1*<br>*ROBO1*<br>*RORC*<br>*RP1-170O19.20*<br>*RP11-480I12.4*<br>*RP11-766F14.2*<br>*RPS6KA3*	

RPUSD2<br>*RRAGD*<br>*RRM2*<br>*RRP1B*<br>*RSF1*<br>*RSPO2*<br>*RTCA*<br>*RUFY3*<br>*RUNX1T1*<br>*S100A8*<br>*SALL3*<br>*SALL4*<br>*SAMD12*<br>*SAP30L*<br>*SBK1*<br>*SBNO1*<br>*SCARA3*<br>*SCD*<br>*SCN11A*<br>*SCN4B*<br>*SCN5A*<br>*SCN8A*<br>*SCRT2*<br>*SCUBE3*<br>*SCYL3*<br>*SDK1*<br>*SEC14L1*<br>*SEC14L5*<br>*SEC16B*<br>*SEC31B*<br>*SECISBP2L*<br>*SEMA4C*<br>*SEMA4F*<br>*SEMA4G*<br>*SENP2*<br>*SENP5*<br>*SESN3*<br>*SESTD1*<br>*SFMBT1*<br>*SFT2D3*<br>*SH2B3*<br>*SH3RF3*<br>*SKIDA1*<br>*SKIL*<br>*SLC10A6*<br>*SLC10A7*<br>*SLC12A9*<br>*SLC16A10*<br>*SLC16A14*	

SLC16A9
SLC1A4
SLC20A1
SLC22A23
SLC25A18
SLC25A24
SLC25A27
SLC25A32
SLC25A4
SLC26A9
SLC2A12
SLC30A1
SLC30A4
SLC30A6
SLC30A7
SLC31A1
SLC35D2
SLC38A9
SLC45A4
SLC4A4
SLC52A3
SLC5A6
SLC5A9
SLC6A1
SLC6A15
SLC6A5
SLC7A14
SLC8A2
SLC9A9
SLCO5A1
SLK
SMAD2
SMARCAD1
SMARCC1
SMC1A
SMCR8
SMIM13
SMIM3
SMUG1
SNAP23
SNN
SNX1
SNX16
SNX17
SNX30
SNX6
SOCS1
SOCS4
SOCS7

SOST
SOWAHA
SOX13
SOX6
SP8
SPATA13
SPATA2
SPEG
SPIRE1
SPOCD1
SPRED3
SPRYD4
SPTBN4
SRD5A3
SREBF2
SREK1
SREK1IP1
SRGAP1
SRGAP3
SSH1
ST3GAL1
ST8SIA1
STAB2
STARD13
STARD3NL
STARD9
STAT3
STEAP3
STK24
STK40
STOX2
STRBP
STRN
STX17
STX3
STXBP5
STYX
SUB1
SUCLG2
SULF1
SULF2
SURF4
SUV39H2
SWT1
SYNCRIP
SYNJ2BP
SYT1
SYT11
SYT2

SYT7
TAB2
TAF9B
TAOK1
TARBP2
TBKBP1
TBX5
TEAD3
TECPR2
TENM2
TET2
TET3
TEX261
TFR2
TGDS
TGFBR1
TGFBR3
THAP9
THBS1
THOC2
THRA
THRSP
TIA1
TIMM17B
TMC7
TMED5
TMEM110
TMEM135
TMEM143
TMEM167A
TMEM178B
TMEM194A
TMEM2
TMEM233
TMEM234
TMEM251
TMEM255A
TMEM26
TMEM41A
TMEM65
TMOD2
TMPPE
TMPRSS2
TMTC3
TMX4
TNFAIP3
TNFRSF1B
TNFSF9
TNIK

TOB2
TOR1AIP2
TP53
TPP1
TRABD
TRANK1
TRHDE
TRIB1
TRIB2
TRIM41
TRIM67
TRIM71
TRIOBP
TRPM6
TSC1
TSC22D2
TSPAN18
TSPAN2
TSPEAR
TSPYL5
TTC31
TTC39C
TTL
TTLL4
TUSC2
TXLNA
TXLNG
UBE2G2
UBN2
UFM1
UGCG
UGGT1
UHRF2
ULK2
UNC5A
USP12
USP21
USP24
USP32
USP38
USP44
USP47
USP49
USP6
UTP15
UTRN
VANGL2
VASH2
VAV3

VCPIP1	
VEZT	
VGLL3	
VPS26B	
VSNL1	
VSTM5	
WAPAL	
WARS2	
WASL	
WDFY2	
WDFY4	
WDR37	
WIPI2	
WNK3	
WNT1	
WNT9A	
WNT9B	
XK	
XKR4	
XKR7	
XKR8	
XKRX	
XPOT	
XRN1	
XYLT1	
YAF2	
YBX2	
YOD1	
YPEL2	
YTHDF3	
YY1	
ZBTB10	
ZBTB16	
ZBTB26	
ZBTB37	
ZBTB39	
ZBTB5	
ZBTB8B	
ZC3H10	
ZC3H3	
ZC3HAV1L	
ZCCHC11	
ZCCHC3	
ZFYVE26	
ZKSCAN5	
ZNF10	
ZNF200	
ZNF202	
ZNF24	

ZNF275
ZNF280B
ZNF282
ZNF322
ZNF341
ZNF354A
ZNF354B
ZNF362
ZNF391
ZNF451
ZNF462
ZNF473
ZNF512B
ZNF516
ZNF566
ZNF583
ZNF620
ZNF641
ZNF644
ZNF652
ZNF689
ZNF697
ZNF70
ZNF710
ZNF74
ZNF740
ZNF774
ZNF783
ZNF784
ZNF879
ZPLD1
ZSWIM4
ZSWIM5

Table S11. Balance diagnostics before and after inverse propensity score weighting

	Absolute weighted standardized difference (%)			
	Means	Higher order moments*	Interactions†	Kolmogorov smirnov test statistic
Model A				
Before weighting	32% - 72%	33% - 66%	23% - 86%	0.23- 0.42
After weighting	4% - 27%	0.2% - 15%, except 30% for Age	0.7% - 28%	0.13 - 0.31
Improvement in balance?	Yes	Yes	Yes	Yes
Model B				
Before weighting	11% - 70%	9% - 59%	21% - 76%	0.13 - 0.39
After weighting	0.1% - 12%, except 24% for Age	0.1% - 8%, except 26% for Age	0.2% - 21%	0.14 - 0.30
Improvement in balance?	Yes	Yes	Yes	Yes

Model A, IS and SS; Model B, IS+ and SS+.

*Only second order moments (squares) assessed.

†Only two-way interactions assessed.

Supplemental Figures

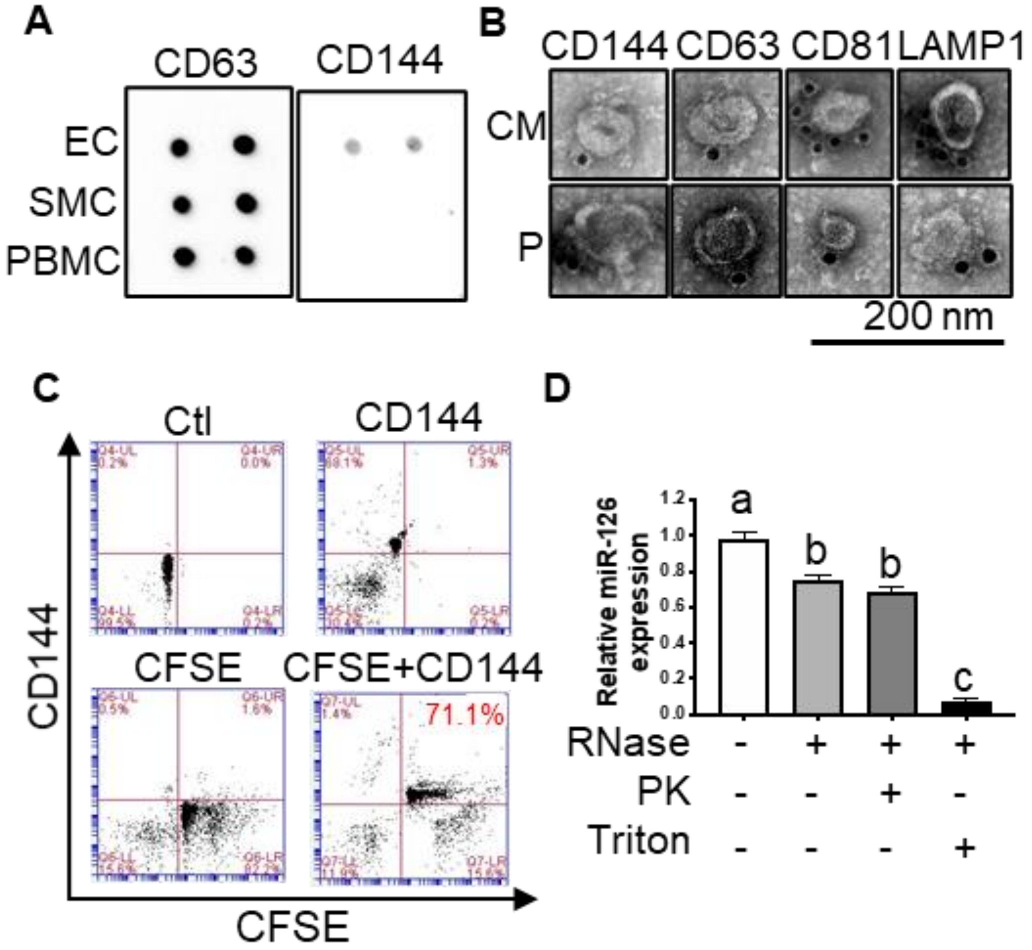

Figure S1. Characterizations of EC-EVs. A, Dot blot immunostaining of CD63 and CD144 in EVs collected from conditioned media of endothelial cells (EC), smooth muscle cells (SMC), and peripheral blood mononuclear cells (PBMC). N=2 replicates each, equal protein loading per spot. **B,** Immunogold staining of EVs from EC-conditioned media (CM) and human plasma (P) with EV-specific markers (CD81, CD63, and LAMP1) and EC-surface marker (CD144) on EC-EVs. **C,** Flow cytometry shows the presence of $CD63^+CD144^+$ EVs in human plasma samples using magnetic beads conjugated with anti-CD63 antibody and co-stained with anti-CD144 antibody. CFSE dye was used to labeled lipid-based EVs. **D**, Protection assay demonstrates the ability of EC-EVs isolated from EC conditioned media to protect miR-126. Expressional levels of miR-126 in each group was normalized to Mock. Ctl: beads incubated with plasma without stains; PK: protein kinase.

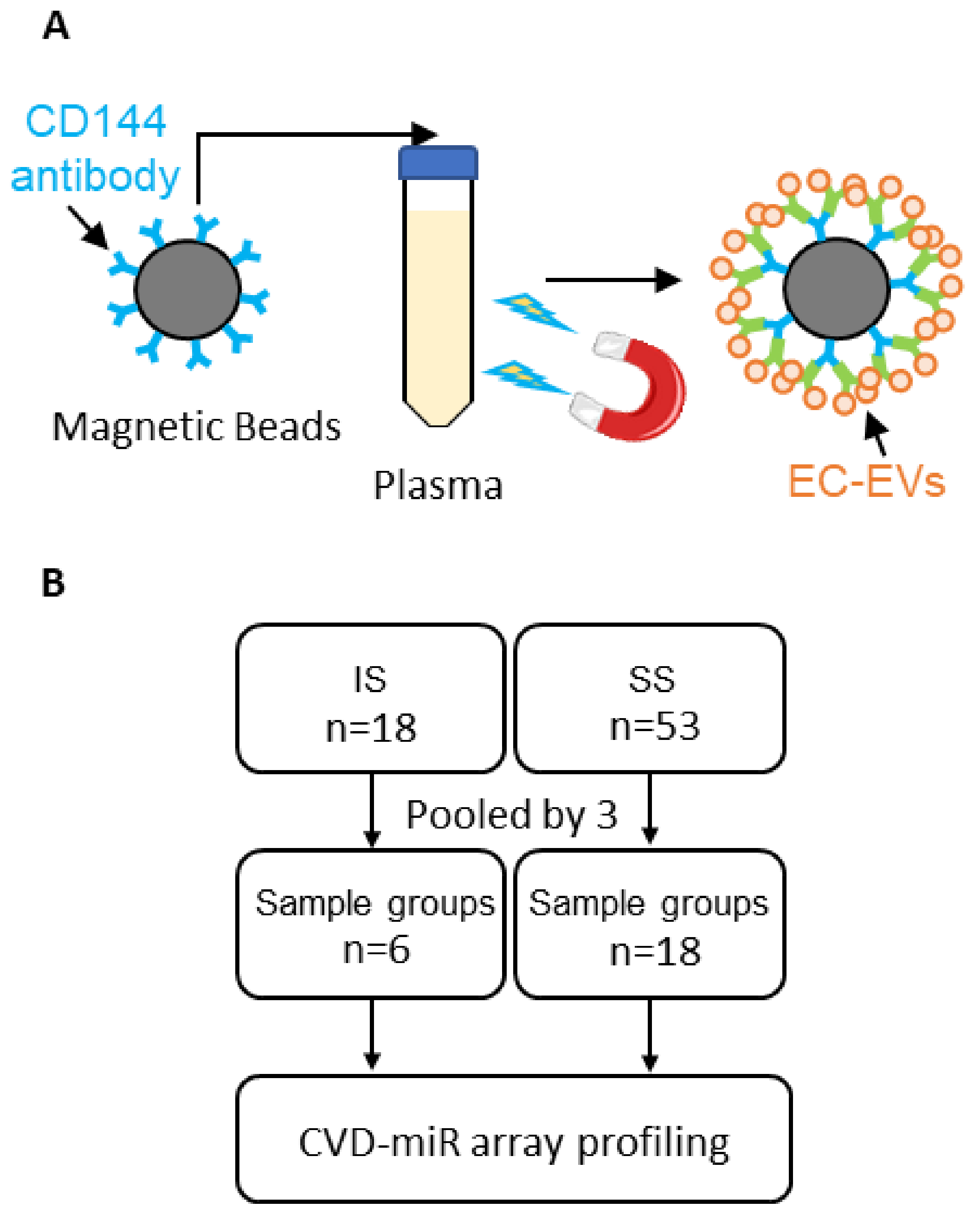

Figure S2. Strategy of cardiovascular disease (CVD)-related miR profiling from IS and SS groups. A, Schematic protocol of EC-EV immunoprecipitation from plasma. **B,** Schematic protocol of EC-EV miR analysis and CVD-miR profiling (Human Cardiovascular Disease Array, Qiagen) using pooled RNA samples from IS and SS groups.

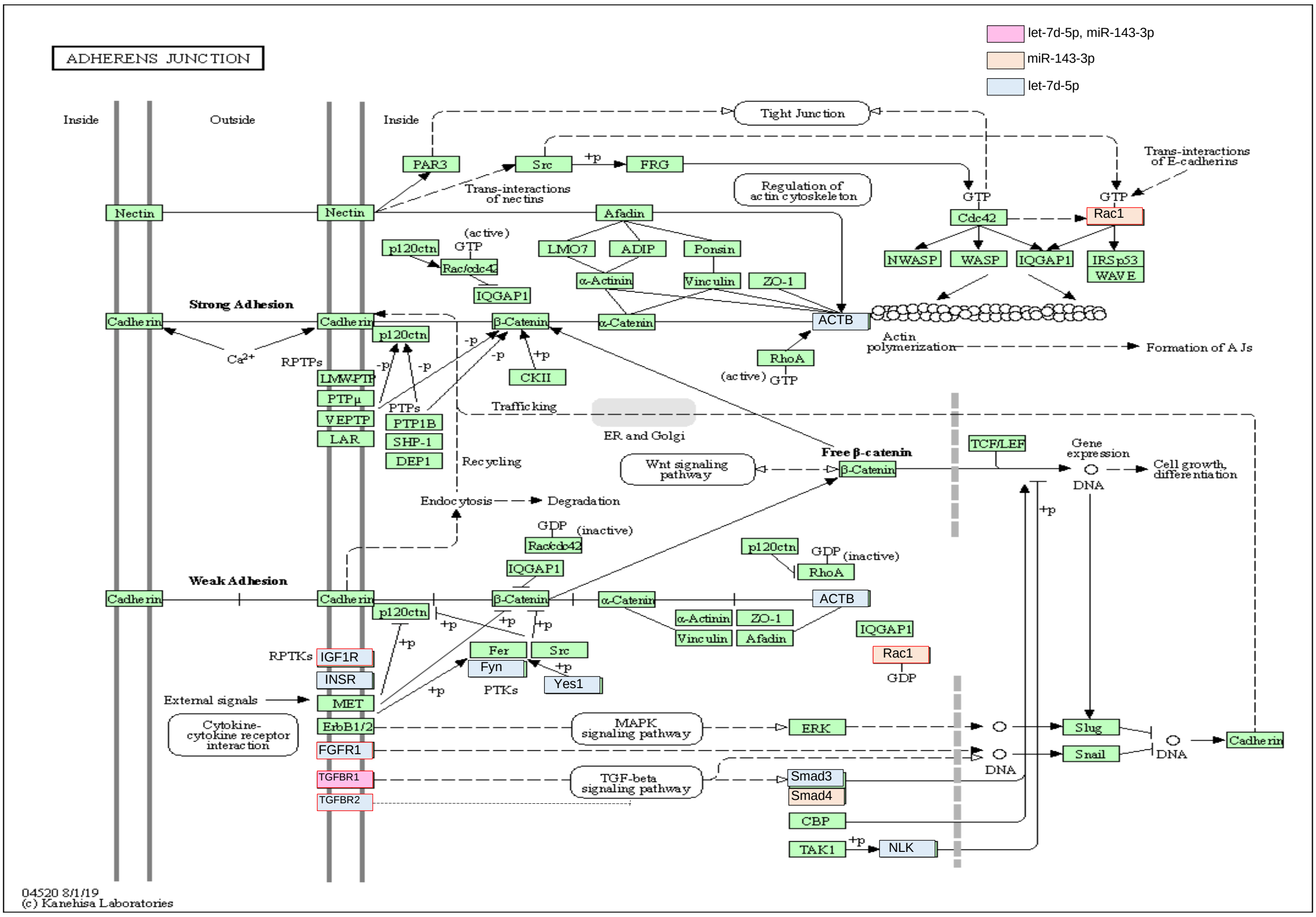

Figure S3. Molecular map of KEGG adherens junction pathway highlighting sitting time-associated miR target genes. Adherens junction pathway components targeted by let-7d-5p or/and miR-143-3p are color-coded (see legend). Red outline indicates components that are direct targets of let-7d-5p and/or miR-143-3p, black outline indicates components that are indirect targets. KEGG pathway image (map04520) is used with permission from KEGG.[1]

[1] Kanehisa M, Sato Y, Kawashima M, Furumichi M, Tanabe M. KEGG as a reference resource for gene and protein annotation. Nucleic Acids Res. 2016 Jan 4;44(D1):D457-62. doi: 10.1093/nar/gkv1070.
